# CDK4-selective inhibitor AU2-94 for the treatment of advanced and therapy-resistant prostate cancer

**DOI:** 10.1186/s13046-026-03763-x

**Published:** 2026-06-17

**Authors:** Ava Safaroghli-azar, Travis Croft, Laychiluh B. Mekonnen, Jimma Lenjisa, Maja Dickel, Raj K. Shrestha, Samyukta Sita, Nicholas Choo, Michael Roach, Sunita K.C. Basnet, Mitchell G. Lawrence, Luke A. Selth, Shudong Wang

**Affiliations:** 1 Drug Discovery and Development, School of Pharmacy and Biomedical Sciences, College of Health, Adelaide University, Adelaide, SA 5001 Australia; 2 Flinders University, College of Medicine and Public Health, Flinders Health and Medical Research Institute, Bedford Park, SA 5042 Australia; 3 Melbourne Urological Research Alliance, Biomedicine Discovery Institute, Monash University, Clayton, VIC 3800 Australia; 4 Peter MacCallum Cancer Centre, Melbourne, VIC 3000 Australia; 5 Sir Peter MacCallum Department of Oncology, The University of Melbourne, Melbourne, VIC 3010 Australia; 6 Cabrini Institute, Cabrini Health, Malvern, VIC 3144 Australia; 7 College of Health, Adelaide University, Adelaide, SA 5001 Australia

**Keywords:** CDK4 inhibitor, AU2-94, atirmociclib, prostate cancer, Docetaxel, Enzalutamide, CRPC

## Abstract

**Introduction:**

CDK4-selective inhibitors are emerging as promising anticancer agents. Relative to dual CDK4/6 inhibitors, CDK4-selective inhibitors have the potential to retain efficacy while improving tolerability. However, the therapeutic value and mechanistic consequences of selectively targeting CDK4 in prostate cancer remain largely undefined. Here, we investigated the anti-tumour activity and mode of action of the CDK4 inhibitors AU2-94 and atirmociclib across diverse prostate cancer models.

**Methods:**

Antiproliferative activity was assessed in a panel of AR-driven and AR-independent prostate cancer cell lines spanning hormone-sensitive and castration-resistant states. Efficacy was further evaluated in organoids derived from patient-derived xenografts and in xenograft mouse models. Biochemical and molecular analyses were performed to evaluate CDK4 selectivity, RB pathway engagement, transcriptional reprogramming, and downstream effects on cell-cycle regulation, and resistance-associated programmes. AU2-94 was also tested in combination with standard-of-care therapies (enzalutamide and docetaxel) and the PI3K inhibitor alpelisib.

**Results:**

AU2-94 exhibited greater selectivity for CDK4 compared with atirmociclib. AU2-94 suppressed proliferation across prostate cancer models irrespective of AR status and retained activity in aggressive and therapy-resistant settings. In RB-proficient in vitro models, AU2-94 reduced RB phosphorylation, attenuated E2F1-dependent transcriptional outputs, activated AR signalling, and decreased expression of proliferation-associated factors such as FOXM1. In vivo, AU2-94 inhibited the growth of both AR-driven (LNCaP) and AR-independent (PC3) xenografts and suppressed RB pathway signalling. Moreover, AU2-94 demonstrated additive or synergistic effects when combined with enzalutamide, docetaxel, or alpelisib that were associated with reinforced cell-cycle blockade and suppression of resistance-associated signalling.

**Conclusion:**

These findings establish selective CDK4 inhibition as a therapeutically active and mechanistically rational strategy in prostate cancer and support AU2-94 as a candidate for further preclinical and clinical development, including in combination regimens for advanced and therapy-resistant disease.

**Supplementary Information:**

The online version contains supplementary material available at 10.1186/s13046-026-03763-x.

## Introduction

Prostate cancer is the second most common non-cutaneous malignancy and the fifth leading cause of cancer-related mortality in men worldwide [1, 2]. The disease originates from prostate luminal epithelial cells that exhibit aberrant androgen receptor (AR) signalling, which drives proliferation and survival [3, 4]. Accordingly, androgen deprivation therapy and AR signalling inhibitors remain the cornerstone of treatment for advanced and metastatic disease [5]. Unfortunately, these AR-targeted therapies are not curative, and tumours eventually develop therapy-resistant phenotypes [3]. This stage of the disease, known as castration-resistant prostate cancer (CRPC), is highly heterogeneous, encompassing both AR-driven and AR-independent phenotypes and is, in most cases, rapidly lethal [6, 7]. Therapeutic options for CRPC include additional AR-targeted therapies, taxane-based chemotherapies, and radiopharmaceuticals [8–11]. While these treatments prolong survival, their efficacy is limited [8, 12, 13]. Therefore, there is a critical need to identify new therapeutic strategies that target alternative cancer-causing pathways.

From early-stage prostate cancer to advanced CRPC, AR signalling and cooperating oncogenic pathways converge on cell-cycle regulatory proteins, particularly D-type cyclins and their catalytic partners, cyclin-dependent kinase (CDK) 4 and CDK6, to support tumour growth [14]. Cyclin D-CDK4/6 complexes phosphorylate the retinoblastoma (RB) protein, relieving repression of E2F transcription factors and promoting G1/S cell-cycle progression [14, 15]. Dysregulation of this axis, such as elevated cyclin D1 expression, is associated with aggressive clinicopathological features, resistance to AR-targeted therapies, metastasis, and poor survival outcomes in prostate cancer patients [16–19], providing a strong biological rationale for therapeutic CDK4/6 inhibition.

Dual CDK4/6 inhibitors – palbociclib, ribociclib and abemaciclib – provide substantial benefit to oestrogen receptor-positive breast cancer patients when combined with endocrine therapy [20]. However, the clinical success of these inhibitors has not translated into comparable efficacy in prostate cancer [21]. A major challenge underlying these outcomes is dose-limiting haematologic toxicity, which restricts dosing intensity and complicates rational combination strategies [22–24]. Emerging evidence suggests that these toxicities are largely driven by CDK6 inhibition within the haematopoietic compartment. Genetic studies have demonstrated that, whereas *Cdk6* is essential for haematopoietic stem cell activation and differentiation, *Cdk4* is comparatively dispensable in normal haematopoiesis [25, 26]. This functional divergence has provided a strong biological rationale for the development of selective CDK4 inhibitors. Despite these advances, a key unresolved question is whether dual CDK4/6 inhibition is biologically required in prostate cancer or whether targeting CDK4 alone may achieve sufficient tumour suppression.

Recently, CDK4-selective inhibitors have emerged as a next-generation therapeutic strategy [20]. Among these, AU2-94, a first-in-class CDK4-selective inhibitor, has demonstrated anti-tumour activity across a range of preclinical models, including ovarian, breast, colorectal, and glioblastoma [27, 28], and has shown pharmacological myeloid protection in RB-negative triple-negative breast cancer [29]. Atirmociclib (PF-07220060) represents the most clinically advanced agent in this class and is currently undergoing evaluation across multiple tumour types [30]. Additional CDK4-selective inhibitors are also progressing through early clinical development, including BGB-43,395, which is being assessed in breast cancer and other solid tumours [31], and RGT-587 (GDC-0587), which is under phase I investigation in patients with breast cancer previously treated with CDK4/6 inhibitors (NCT07214662).

Despite considerable interest in selective CDK4 targeting, key mechanistic and translational questions remain unresolved. At present, limited information is available regarding the biochemical selectivity profiles, pharmacokinetic properties, and preclinical efficacy of these agents. Moreover, characterization of kinase specificity across divergent assay platforms has raised questions about the true degree of CDK4 selectivity and the extent of off-target engagement within this emerging class. Subtle differences in kinase selectivity may translate into distinct effects on cell-cycle regulation, mRNA transcription, tumour dependency states and, ultimately, clinical positioning. Accordingly, rigorous comparative evaluation is required to define mechanistic distinctions and therapeutic implications.

In this study, we sought to determine whether selective CDK4 inhibition is sufficient to suppress prostate tumour growth and whether CDK4 targeting constitutes a rational mechanistic strategy in this disease context. To this end, we performed a comprehensive head-to-head biochemical, cellular, and in vivo comparison of AU2-94 with atirmociclib and dual CDK4/6 inhibitors. These analyses delineate differences in CDK selectivity, downstream signalling consequences, and tumour dependency across prostate cancer models.

Our integrated studies support the therapeutic utility of CDK4 inhibition in prostate cancer and demonstrate the robust anti-tumour activity of AU2-94 across aggressive prostate cancer models, both as monotherapy and in combination settings.

## Materials and methods

### Chemicals

AU2-94 was prepared by Changzhou LeSun Pharmaceuticals Ltd. (Changzhou, China) using synthetic procedures adapted from previously reported methods [32]. Atirmociclib, enzalutamide, alpelisib, palbociclib, ribociclib, and abemaciclib were purchased from MedChemExpress (New Jersey, USA). Docetaxel was obtained from the Royal Adelaide Hospital Pharmacy (Adelaide, Australia). All secondary antibodies were from Cell Signaling Technology (Danvers, MA, USA), and AlphaLISA kits were obtained from PerkinElmer (Victoria, Australia). The sources of primary antibodies are listed in Supplementary Table S1.

### Cell culture

Prostate cancer cell lines, including LNCaP, 22Rv1, C4-2B, PC3, and DU145, were obtained from the American Type Culture Collection (ATCC). The enzalutamide-resistant cell lines MR42D and MR49F, along with their parental line V16D [33], were generously provided by Professor Amina Zoubeidi (University of British Columbia, Canada). LNCaP, 22Rv1, C4-2B, PC3, and DU145 cells were maintained in RPMI-1640, DMEM, or EMEM media supplemented with 10% foetal bovine serum (FBS) under standard culture conditions (37 °C, 5% CO₂, in a humidified incubator). MR42D and MR49F cells were cultured in RPMI-1640 medium supplemented with 10% FBS and 10 µM enzalutamide.

### Cell proliferation assays

The antiproliferative effects of selective CDK4 inhibitors, dual CDK4/6 inhibitors, and combination treatments were evaluated using the MTT (3-(4,5-dimethylthiazol-2-yl)-2,5-diphenyltetrazolium bromide) assay. Prostate cancer cell lines were seeded in 96-well plates at a density of 0.6-1.0 × 10³ cells per well and treated with increasing concentrations of the indicated compounds for 144 h. For sequential treatment experiments in C4-2B cells, cells were initially exposed to AU2-94 for 72 h. The medium was then replaced with fresh medium containing enzalutamide at the indicated concentrations, and incubation was continued for an additional 72 h. At the end of the treatment period, cell viability was assessed by adding MTT reagent (Thermo Fisher Scientific, Victoria, Australia) to each well. Following incubation, the resulting formazan crystals were dissolved in DMSO, and absorbance was measured at 570 nm using a PerkinElmer plate reader (PerkinElmer, Buckinghamshire, UK).

Dose–response curves were generated by plotting the percentage of viable cells against the logarithm of compound concentrations using GraphPad Prism (version 10.6.1; GraphPad Software, CA, USA). Growth inhibition 50 (GI₅₀) values were determined by nonlinear regression analysis using a four-parameter variable-slope model. Drug interactions were determined by calculating the combination index (CI) using the Chou-Talalay method [34] through CompuSyn software (ComboSyn, New Jersey, USA), where CI values of < 1, 1, and > 1 were indicative of synergistic, additive, and antagonistic interactions, respectively.

### Cell cycle analysis

To evaluate the impact of treatment on cell-cycle progression, prostate cancer cells were treated with test compounds as single agents or in combination for 24–48 h. Following treatment, cells were harvested by trypsinisation, washed with phosphate-buffered saline (PBS), and fixed by the dropwise addition of ice-cold 70% ethanol. Samples were incubated on ice for 15 min. After centrifugation at 300 × *g* for 5 min, the supernatant was discarded, and the cell pellets were resuspended in propidium iodide (PI) staining buffer containing 50 µg/mL PI, 0.1 mg/mL RNase A, and 0.05% Triton X-100. Cells were incubated in the dark at room temperature for 1.5 h to allow DNA staining. Cell-cycle distribution was analysed using a CytoFLEX flow cytometer (CytExpert, Beckman Coulter, California, USA), and data were analysed using CytExpert version 2.1 software (Beckman Coulter, California, USA).

### Colony formation assay

To assess the effect of treatment on clonogenic growth, prostate cancer cells were seeded in 6-well plates and treated with compounds as single agents or in combination for 10 days. The medium was refreshed every 72 h to maintain drug exposure according to the treatment schedule. After 10 days, the medium was aspirated, and wells were gently washed with PBS. Colonies were fixed and stained using a crystal violet solution (0.05% crystal violet, 1% formaldehyde, 1% PBS, and 1% methanol). After staining, plates were rinsed with distilled water and air-dried. Colony images were acquired, and the number of colonies was quantified using ImageJ software (National Institutes of Health, MD, USA). Clonogenic inhibition was expressed as a percentage relative to untreated control cells.

### Western blotting

To assess changes in protein expression, prostate cancer cells were exposed to increasing concentrations of AU2-94 and atirmociclib for 24 h. Following treatment, cells were lysed, and total protein concentrations were quantified using the Bio-Rad DC protein assay (Bio-Rad, New South Wales, Australia), according to the manufacturer’s instructions. Equal amounts of protein (20 µg per sample) were denatured by heating and resolved by SDS-PAGE on 4–20% polyacrylamide gradient gels. Proteins were subsequently transferred onto nitrocellulose membranes, which were blocked in 5% skimmed milk (SM) prepared in Tris-buffered saline containing 0.1% Tween-20 (TBS-T) for 1 h at room temperature. Membranes were incubated overnight at 4 °C with primary antibodies. A list of primary antibodies is provided in Supplementary Table S1. After washing with TBS-T, membranes were incubated with horseradish peroxidase (HRP)-conjugated secondary antibodies (Cell Signaling Technology, Danvers, MA, USA) for 1 h at room temperature. Immunoreactive bands were visualised using Amersham ECL Prime/Select Western blotting detection reagent (GE Healthcare, New South Wales, Australia) and imaged using the ChemiDoc™ MP Imaging System (Bio-Rad, New South Wales, Australia). Band intensities were quantified using ImageJ software (NIH, MD, USA), and protein expression levels were normalised to a housekeeping protein.

### AlphaLISA^®^ sureFire^®^ ultra™ assays

Protein levels were measured using AlphaLISA^®^ SureFire^®^ Ultra™ assays (PerkinElmer, Victoria, Australia). Prostate cancer cells were treated with inhibitors as single agents or in combination, according to the designated treatment schedule. Following treatment, cells were lysed using 1× AlphaLISA lysis buffer, and protein concentrations were determined using the Bio Rad DC protein assay. Protein lysates were then transferred to 384-well white assay plates. Each well was incubated with the AlphaLISA Acceptor Mix, comprising reaction buffer 1 (1:2.1), reaction buffer 2 (1:2.1), activation buffer (1:25), and acceptor beads (1:50), for 1 h at room temperature. Subsequently, the AlphaLISA Donor Mix, containing donor beads diluted 1:50 in dilution buffer, was added, and the plates were incubated for a further 2 h in the dark. Luminescence was measured using an Alpha-enabled plate reader (Victor Nivo, Multilabel plate reader). Protein expression levels were normalised to total protein concentration in each lysate and expressed as a percentage relative to untreated control cells. Basal expression levels for each cell line were further normalised to the corresponding housekeeping protein, cofilin.

### Biochemical assays

The kinase inhibition profiles of AU2-94 and atirmociclib were assessed using the ADP-Glo™ assay (Promega, New South Wales, Australia). Serial dilutions of each compound were prepared and transferred to a 384-well white microplate, followed by the addition of 2 µL of kinase master mix to each well. Plates were incubated for 90 min at room temperature. For CDK6/cyclin D3 assays, plates were first incubated at 37 °C with 5% CO₂ for 20 min, followed by an additional 10-minute incubation at room temperature. Subsequently, ADP-Glo reagent was added and incubated for 40 min to deplete unconsumed ATP. This was followed by the addition of kinase detection reagent, with a further incubation for 30–40 min in the dark, depending on the kinase. Staurosporine (40 µM) and 1% DMSO were included as positive and negative controls, respectively. Luminescence was measured using a Victor Nivo™ multimode plate reader (PerkinElmer, Victoria, Australia). Signals were normalised to the positive and negative controls to calculate the percentage of residual kinase activity. Half-maximal kinase inhibitory concentrations (IC₅₀ values) were determined by plotting residual activity against the logarithm of compound concentrations and fitting a four-parameter nonlinear regression curve using GraphPad Prism (version 10; GraphPad Software, CA, USA). Inhibition constants (*K*_i_) were calculated from IC_50_ values using the Cheng-Prusoff equation [35]. Each kinase assay was conducted at its respective *K*_m_ (ATP) concentration. Detailed compositions and reaction conditions for each CDK/cyclin complex are provided in the Supplementary Methods.

### In vivo efficacy studies

All animal procedures were approved by the University of South Australia Animal Ethics Committee (Ethics Approval Numbers: U12-23 and U33-22) and conducted in accordance with institutional guidelines for the care and use of laboratory animals.

For both LNCaP and PC3 xenograft models, male nude (nu/nu) BALB/c mice (4–6 weeks old) were used. LNCaP and PC3 cells (5 × 10⁶) were resuspended in 100 µL of a 1:1 mixture of growth factor-reduced Matrigel (Corning, Australia) and serum-free RPMI-1640 medium and injected subcutaneously into the right flank of each mouse. Once tumours reached volumes of 50–250 mm³, mice were randomised into treatment groups. In the LNCaP model, mice (*n* = 5 per group) received either vehicle (distilled water, PO BID) or AU2-94 (75 mg/kg, PO BID) for 21 days. In the PC3 model, mice were treated for 21 days with vehicle (distilled water, PO BID; *n* = 10), AU2-94 (15, 37.5, or 50 mg/kg, PO BID; *n* = 10 per group), ribociclib (37.5 mg/kg, PO BID; *n* = 7), docetaxel (10 mg/kg, IP QW; *n* = 10), or the combination of AU2-94 (37.5 mg/kg, PO BID) and docetaxel (10 mg/kg, IP QW; *n* = 10). In a separate study, PC3 tumour-bearing mice were treated with vehicle (distilled water, PO BID; *n* = 10), AU2-94 (25 mg/kg, PO BID; *n* = 10), or atirmociclib at an equimolar dose (23 mg/kg, PO BID; *n* = 10) to compare the effects of these two inhibitors.

Mice were monitored daily for clinical signs of toxicity and changes in body weight using a Clinical Record Scoring (CRS) system. Clinical assessments included monitoring for dehydration, diarrhoea, abnormal breathing, skin tenting, seizures, ruffled coats, body hunching, and reluctance to move. The CRS assessment criteria are provided in the Supplementary Methods. Tumour volume was measured every other day prior to dosing using callipers.

In accordance with the approved ethical protocols (U12-23 and U33-22), a maximum tumour volume of 2000 mm³ was established as the predefined experimental endpoint for all animal studies in this manuscript. We confirm that this maximal tumour size was not exceeded in any of the animal studies presented in this manuscript. Animals were euthanized when tumour volume exceeded 2000 mm³ or when body weight loss exceeded 15% relative to baseline. The percentage of tumour volume ratio (T/C) was calculated as previously described [36].

### Analysis of public transcriptomic data

Transcriptomic data from The Cancer Genome Atlas (TCGA, Firehose Legacy) [37] were downloaded via the cBioPortal platform [38]. Transcriptomic data from Grasso et al. [39] were obtained from the Gene Expression Omnibus (GEO; GSE35988). Survival analyses were performed using cBioPortal.

### Analysis of CDK4 and CDK6 dependencies across cancer types using DepMap

To assess CDK4 and CDK6 essentiality across diverse cancer types, gene dependency data were obtained from the DEMETER2 (Achilles + DRIVE + Marcotte) Gene Dependency Score dataset, accessed through the Cancer Dependency Map (DepMap) portal (https://depmap.org). This dataset integrates genome-scale RNA interference (RNAi) viability screens performed across a large panel of human cancer cell lines, including those derived from bone (*n* = 16), prostate (*n* = 7), breast (*n* = 74), pleura (*n* = 6), central nervous system/brain (*n* = 45), head and neck (*n* = 20), soft tissue (*n* = 18), pancreas (*n* = 32), ovary/fallopian tube (*n* = 36), uterus (*n* = 21), lung (*n* = 126), skin (*n* = 48), liver (*n* = 18), kidney (*n* = 31), oesophagus/stomach (*n* = 48), bowel (*n* = 48), cervix (*n* = 4), bladder/urinary tract (*n* = 14), eye (*n* = 3), peripheral nervous system (*n* = 9), thyroid (*n* = 4), lymphoid (*n* = 26) and myeloid (*n* = 21) [40].

### RNA sequencing

LNCaP cells were seeded in 6-well plates at a density of 4 × 10^5^ per well (24 h treatment) or 1.5 × 10^5^ per well (72 h treatment). After 24 h, cells were treated with AU2-94, atirmociclib, ribociclib or vehicle control for 24–72 h. Total RNA was extracted using TRIzol reagent. Three biological replicates were generated per treatment condition. RNA libraries were prepared using Universal Plus Total RNA-Seq with NuQuant kits (Tecan Life Sciences) protocol according to the manufacturer’s instructions and included 12 cycles of amplification. Samples were sequenced at the South Australian Genomics Centre (SAGC) on an MGI DNBSEQ G400 with FCL PE100 chemistry.

Raw FASTQ files were processed using the nf-core/rnaseq pipeline (v3.18.0) [41]. Reads were aligned to the human reference genome (GRCh38) using STAR (v2.6.1d) [42], and differential gene expression analysis was conducted using DESeq2 [43]. The RNA-sequencing data were deposited in NCBI’s Gene Expression Omnibus (GSE330358).

Gene set enrichment analysis (GSEA) [44] was performed in R (v4.4.3) using ClusterProfiler [45]. Genes were pre-ranked using a signed p-value metric. Gene sets were obtained from the Molecular Signatures Database [46] or from published datasets [47, 48].

### Organoid establishment and culture

PDX 224R-Cx was previously established by the Melbourne Urological Research Alliance (MURAL) [49]. The patient provided written informed consent in accordance with human ethics approvals from Monash Health (RES-20-0000-107 C), Cabrini Hospital (07-07-04-14), and Monash University (1636). The serially transplantable PDX was maintained in castrated NSG mice in accordance with Monash University animal ethics approvals (MUAEC-4 41088).

Organoids were cultured from PDX tissue using previously described methods [49–52]. PDX tissue was finely minced with scalpels and digested for 40 min at 37 °C in RPMI-1640 containing 0.2 mg/ml DNase I (Roche) and 0.65 U/mL Liberase TM (Roche). Digested samples were centrifuged, filtered through 70 μm cell strainers, treated with red blood cell lysis buffer (Sigma-Aldrich), and washed with RPMI containing 5% FCS, 10 U/mL penicillin and 10 µg/mL streptomycin. Cells were embedded in growth factor-reduced, phenol-red free, and IdEV-free Matrigel at a density of 2.5 × 10^4^ cells per 20 µL of Matrigel in 48-well plates. Plates were inverted and incubated at 37 °C to allow the Matrigel to solidify, then re-inverted and cultured in ENR-2 medium as previously described [50–52].

ENR-2 media comprised Advanced DMEM/F12 (Thermo Fisher, Waltham, MA, USA) supplemented with 50 ng/mL epithelial growth factor (Sigma-Aldrich), 5% (v/v) R-spondin 1 (Monash Biomedicine Discovery Institute Organoid Program), 10% (v/v) Noggin (Monash Biomedicine Discovery Institute Organoid Program), 10 ng/mL fibroblast growth factor 10 (VWR International, Queensland, Australia), 5 ng/mL fibroblast growth factor 2 (PeproTech, Rocky Hill, New Jersey, USA), 10 mM nicotinamide (Sigma-Aldrich), 0.5 µM A83-01 (Sigma-Aldrich), 1 × B27 supplement (Thermo Fisher Scientific), 2 mM GlutaMAX (Thermo Fisher Scientific), and 1 µM prostaglandin E2 (Tocris Bioscience, Bristol, UK). During organoid establishment, 10 µM Y-27,632 dihydrochloride (Selleck Chemicals) was also added to the culture medium.

### Organoid viability assay

Organoids were allowed to establish for 48 h before treatment with vehicle control, AU2-94, atirmociclib, or ribociclib at the indicated concentrations for 12 days, with medium replenished every 3–5 days. All treatments were performed in triplicate. Organoids metabolic activity was assessed using the PrestoBlue Cell Viability Reagent (ThermoFisher Scientific) on days 4, 7, and 12. Following removal of the medium and rinsing with PBS, organoids were incubated in PrestoBlue reagent (10% v/v) for 1 h at 37 °C. After incubation, PrestoBlue was aliquoted into clear-bottom 96-well plates (Costar, Washington, DC, USA), with each organoid well distributed into triplicate technical replicates. Fluorescence intensity was measured using a BMG CLARIOstar microplate reader (BMG Labtech, Ortenberg, Germany), with excitation and emission wavelengths set at 535 ± 8 nm and 585 ± 10 nm, respectively. Each PrestoBlue reading was normalised to control wells containing Matrigel only (no organoids) that were incubated with PrestoBlue for 1 h at 37 °C.

### qRT-PCR analysis

Total RNA extraction, cDNA synthesis, and quantitative PCR were performed as previously described [53], except that PCR was carried out using a BioRad CFX Opus 384 system. *GAPDH* and *ACTB* were used for normalisation. Primer sequences are provided in Supplementary Table S2. The comparative threshold cycle (C_T_) method was applied to quantify mRNA levels in each sample.

### Statistical analysis

Unless otherwise specified, all in vitro studies were conducted in at least three independent replicates. Statistical comparisons between two groups were performed using paired or unpaired Student’s *t*-tests, as appropriate. For analyses involving more than two groups, one-way or two-way ANOVA was applied. Where ANOVA indicated significant differences, post hoc multiple comparisons testing was performed using Tukey’s or Dunnett’s tests, as specified in the figure legends.

Kaplan–Meier curves were generated for disease-free survival analyses in the TCGA cohort. Statistical significance between groups was assessed using the log-rank (Mantel–Cox) test. Hazard ratios (HRs) and corresponding confidence intervals (CIs) were estimated using Cox proportional hazards regression. For gene set enrichment analysis (GSEA), significance was defined as *q* < 0.05. For all other analyses, differences were considered statistically significant when *p* ≤ 0.05. Statistical analyses were performed using GraphPad Prism (version 10; GraphPad Software, California, USA).

## Results

### CDK4 inhibition blocks cell proliferation in diverse prostate cancer models

**Fig. 1:**
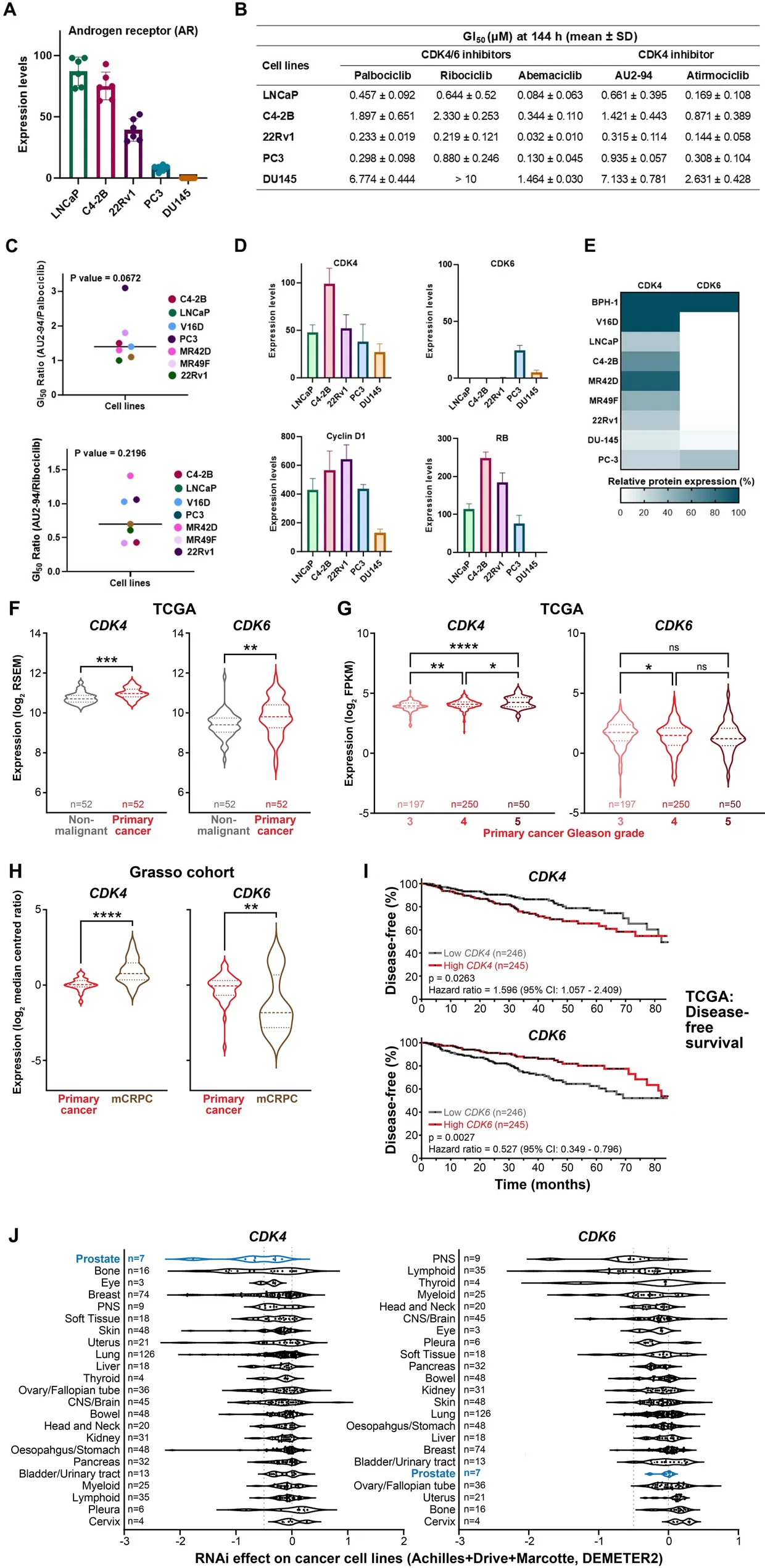
CDK4-dependent proliferation and therapeutic targeting in prostate cancer models. **A** AR expression levels in prostate cancer cells were determined using the AlphaLISA® SureFire® Ultra™ assay. **B** Antiproliferative effect of dual CDK4/6 inhibitors and selective CDK4 inhibitors on prostate cancer cells. **C** Antiproliferative effects of AU2-94 versus palbociclib or ribociclib in prostate cancer cells. Each dot represents a single cell line; the horizontal lines denote the median. **D** and **E** Basal expression levels of CDK4, CDK6, cyclin D1, and RB in prostate cancer cell lines. **F** Expression of CDK4 and CDK6 in localised prostate tumours compared to patient-matched non-malignant tissues in the TCGA cohort [37]. Internal lines in the violin plots demarcate the first quartile (bottom line), median (middle line), and third quartile (top line). Groups were compared using paired *t*-tests. **G** Expression of CDK4 and CDK6 in localised prostate cancer according to increasing Gleason grade in the TCGA cohort. Groups were compared using ANOVA and Tukey’s multiple comparison tests. **H** Expression of CDK4 and CDK6 in localised prostate tumours and metastatic CRPC tumours in the Grasso cohort [39]. Groups were compared using unpaired *t* tests. **I** Kaplan–Meier curves showing disease-free survival probability following radical prostatectomy in patients stratified by the median levels of CDK4 (top graph) or CDK6 (bottom graph) in the TCGA cohort. P values and hazard ratios were determined using log-rank tests. CI, confidence interval. **J** Violin plots of dependency values (DepMap RNAi DEMETER2 scores) for *CDK4* (left) and *CDK6* (right) across 23 tumour types. Each dot represents a cancer cell line. Grey dotted lines show dependency scores of 0 (no dependency) and 1 (gene essentiality). In all panels, p values are: *, p ≤ 0.05; **, p ≤ 0.01; ***, p ≤ 0.001; ****, p ≤ 0.0001; ns, not significant

To evaluate AU2-94 in prostate cancer, we first assessed its antiproliferative effects across a diverse panel of prostate cancer cell lines and benchmarked its activity against approved CDK4/6 inhibitors (palbociclib, ribociclib and abemaciclib) and the clinically advanced CDK4-selective inhibitor atirmociclib [30]. All inhibitors suppressed proliferation across the panel, irrespective of AR status (Fig. 1A and B).

Intact RB signalling is required for response to CDK4/6 inhibitors; consistent with this, the RB-negative DU145 model was largely resistant to most agents. However, abemaciclib and atirmociclib retained measurable activity in DU145 cells, reflected by comparatively low GI₅₀ values (Fig. 1B). DU145 cells express high levels of CDK9 [54], which may contribute to this sensitivity, as both abemaciclib and atirmociclib have been reported to inhibit CDK9/cyclin T1 in addition to CDK4/6 [30, 55] Notably, GI₅₀ ratio (one-sample *t*-tests) revealed no significant differences between AU2-94 and either palbociclib (*p* = 0.0672) or ribociclib (*p* = 0.2196) across these models, suggesting that concomitant CDK6 inhibition does not confer superior antiproliferative activity (Fig. 1C).

Given the comparable activity of dual CDK4/6 and CDK4-selective inhibition, we next examined CDK4 and CDK6 expression across the cell line panel. CDK4 and cyclin D1 were uniformly expressed, whereas CDK6 expression was detected only in PC3 and, to a lesser extent, DU145 cells (Fig. 1D). High CDK4 expression was also observed in additional prostate cancer cell lines V16D, MR49F, and MR42D, while the benign prostate epithelial cell line BPH-1 expressed both CDK4 and CDK6 (Fig. 1E).

We expanded our analyses to transcriptomic profiles from patient tumours. *CDK4* and *CDK6* mRNA levels were elevated in primary prostate cancer relative to non-malignant prostate tissues, but baseline *CDK4* expression was substantially higher than that of *CDK6* (Fig. 1F). Moreover, *CDK4*, but not *CDK6*, showed a positive association with increasing Gleason grade in primary tumours and was further elevated in metastatic disease (Fig. 1G-H). Consistent with an association between *CDK4* expression and an aggressive disease phenotype, higher *CDK4* expression correlated with a shorter disease-free interval in patients undergoing surgery. In contrast, *CDK6* expression was inversely associated with disease progression in this cohort (Fig. 1I). Finally, DepMap analyses supported these findings by demonstrating that prostate cancer cells are highly dependent on CDK4 but not CDK6 (Fig. 1J).

Collectively, these data indicate that selective CDK4 inhibition is sufficient to achieve antiproliferative efficacy comparable to that of dual CDK4/6 inhibition in prostate cancer models, which aligns with the finding that CDK4, but not CDK6, is a dominant driver of proliferation in prostate cancer. In light of these findings, and given the established contribution of CDK6 inhibition to haematopoietic toxicity [56], we focused subsequent studies on the CDK4-selective inhibitors AU2-94 and atirmociclib.

### Comparison of the biochemical and cellular CDK4-targeting profiles of AU2-94 and atirmociclib

**Fig. 2:**
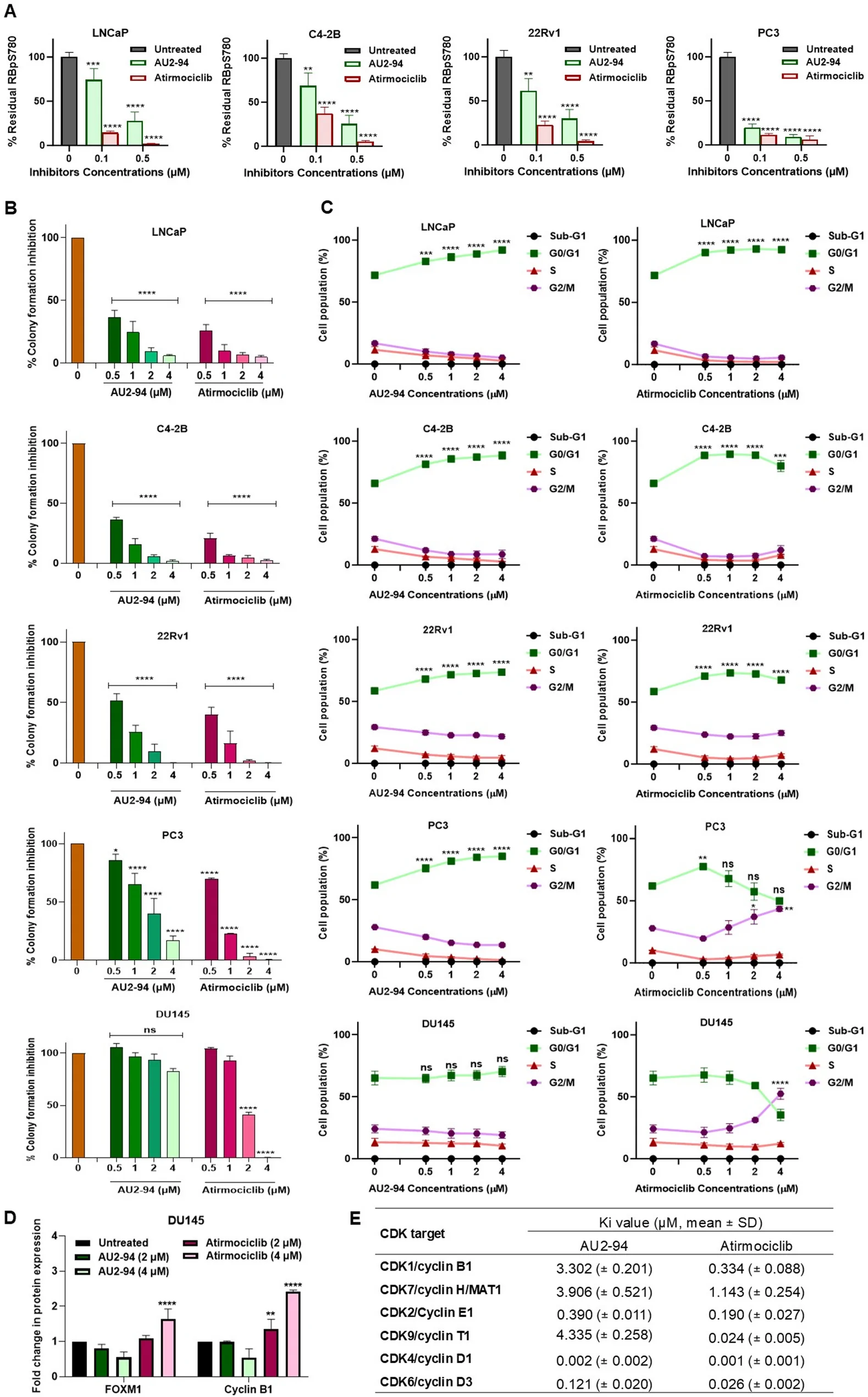
The CDK4-targeting profiles. **A** Phosphorylation of RB (S780) was determined after 24 h of treatment with AU2-94 or atirmociclib using the AlphaLISA® SureFire® Ultra™ assay. **B** Clonogenic growth inhibition of prostate cancer cells was determined after treatment with AU2-94 and atirmociclib for 10 days. Colonies were stained, imaged, and quantified using ImageJ software. Clonogenic inhibition was calculated as a percentage relative to untreated control cells. **C** Changes in cell-cycle distribution induced by 24 h of compound treatment were determined by PI staining. **D** Expression levels of FOXM1 and cyclin B1 in DU145 cells were determined after 24 h of treatment. **E** Compound selectivity against CDK/cyclin complexes was determined using the ADP-Glo kinase assay. Concentration-response curves were generated using a 1:3 serial dilution series with 10 data points for IC₅₀ determination. Inhibition constants (*K*_i_) were calculated from IC₅₀ values using the Cheng–Prusoff equation [35]. Each kinase assay was conducted at its respective *K*_m_ (ATP) concentration. Data are representative of three independent experiments and are presented as mean ± SD. Statistical significance between groups was assessed using one-way ANOVA. Error bars indicate SD. In all panels, p values are: **, p ≤ 0.01; ***, p ≤ 0.001; ****, p ≤ 0.0001; ns, not significant

To further dissect the relative activities and modes of action of CDK4 inhibitors, we conducted a head-to-head comparison of AU2-94 and atirmociclib across a panel of prostate cancer cell lines (LNCaP, C4-2B, 22Rv1, PC3, and DU145). Both compounds suppressed phosphorylation of RB at serine 780 (RBpS780) and significantly impaired clonogenic growth in RB-proficient cell lines (Fig. 2A–B). Interestingly, atirmociclib retained antiproliferative activity in RB-deficient DU145 cells, significantly reducing colony formation at 2 µM and 4 µM (Fig. 2B and Supplementary Fig. S1).

Cell-cycle profiling further distinguished the two compounds. AU2-94 uniformly induced G0/G1 arrest in RB-positive cells but had no detectable effect in DU145 cells (Fig. 2C). By comparison, atirmociclib induced G2/M accumulation in PC3 and DU145 cells (Fig. 2C). Consistent with this observation, high-dose atirmociclib (4 µM) increased expression of cyclin B1 and FOXM1, essential regulators of the G2/M transition (Fig. 2D). In contrast, AU2-94 modestly decreased cyclin B1 and FOXM1 levels in DU145 cells, which aligns with prior reports describing indirect suppression of these factors following CDK4/6 pathway blockade [57, 58].

Because RB-deficient cells bypass the G1/S checkpoint and may rely more heavily on G2/M control, we hypothesised that atirmociclib activity in DU145 cells could reflect off-target inhibition of additional cell-cycle kinases. Kinase profiling supported this prediction, revealing that atirmociclib inhibited CDK1/cyclin B1 (*K*_i_ = 0.334 µM), CDK7/cyclin H/MAT1 (*K*_i_ = 1.143 µM), CDK2/cyclin E1 (*K*_i_ = 0.190 µM), and CDK9/cyclin T1 (*K*_i_ = 0.024 µM) (Fig. 2E). For each of these CDK/cyclin complexes, atirmociclib exhibited substantially greater potency than AU2-94 (Fig. 2E). This broader inhibitory profile, particularly the potent inhibition of CDK9/cyclin T1, likely contributes to atirmociclib activity across prostate cancer cells irrespective of RB status.

We next compared the biochemical potency of AU2-94 and atirmociclib against CDK4/cyclin D1 and CDK6/cyclin D3. Both compounds potently inhibited CDK4, with low nanomolar *K*_i_ values (AU2-94 *K*_i_ = 2 nM; atirmociclib *K*_i_ =1 nM). However, a clear distinction emerged for CDK6: atirmociclib inhibited CDK6 with a *K*_i_ = 26 nM, whereas AU2-94 was substantially less active (*K*_i_ = 121 nM; Fig. 2E). Accordingly, AU2-94 displayed a higher CDK6/CDK4 selectivity ratio (~ 60.5) than atirmociclib (~ 26), indicating more preferential targeting of CDK4 by AU2-94.

These findings are consistent with our prior kinome-wide analysis of 379 kinases, which demonstrated preferential CDK4 targeting by AU2-94 with weaker CDK6 inhibition [27, 28]. Collectively, these direct biochemical and cellular comparisons indicate that AU2-94 has higher selectivity for CDK4 than atirmociclib.

### Cellular pharmacodynamic effects of AU2-94

**Fig. 3:**
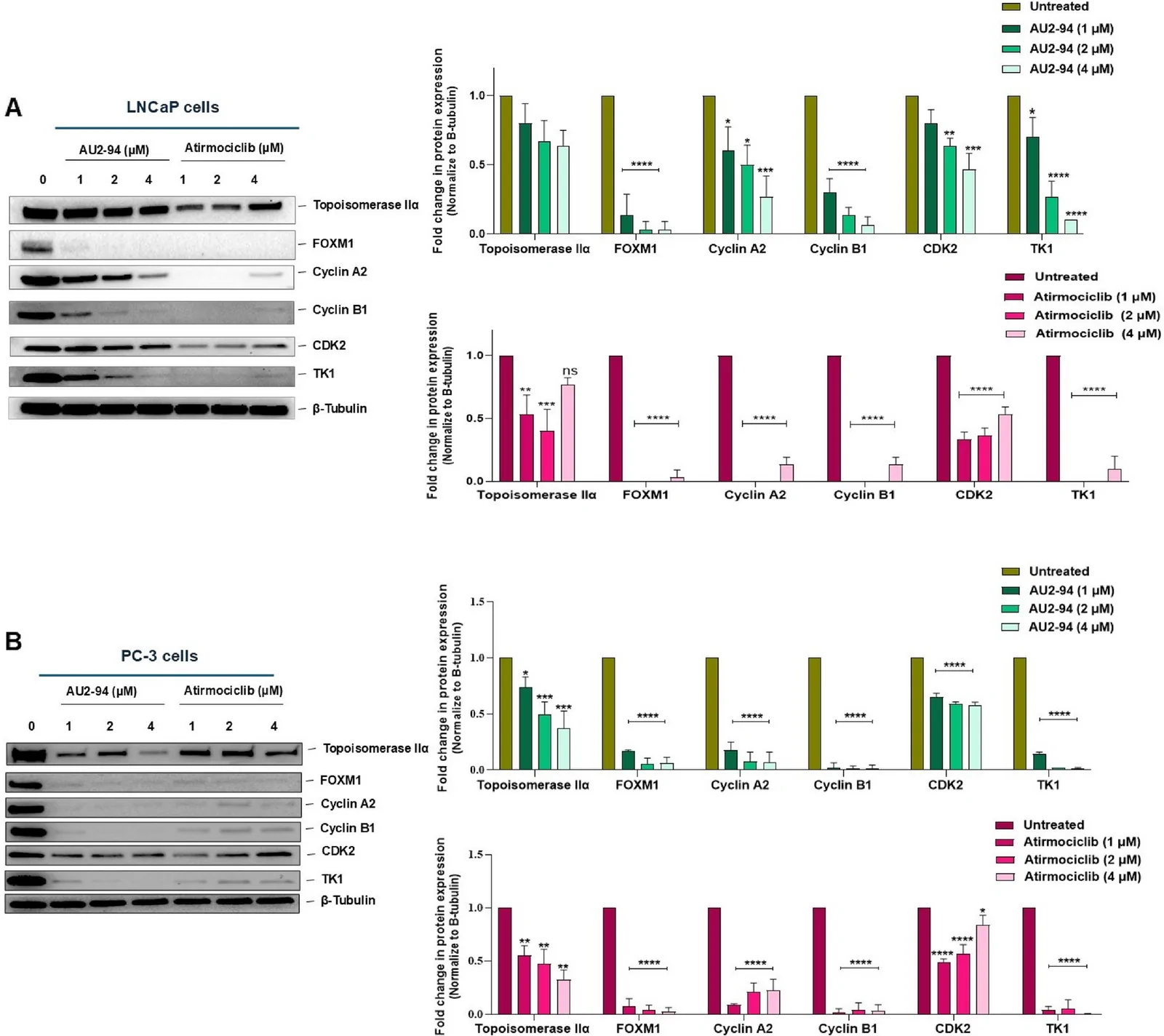
Effect of AU2-94 on LNCaP (**A**) and PC3 (**B**) cells. Both cell lines were treated with increasing concentrations of AU2-94 and atirmociclib for 24 h. Following treatment and preparation of cell lysates, protein expression levels were determined by western blot analysis. Band intensities were quantified using ImageJ software. For each sample, target protein intensity was normalised to the corresponding loading control (β-tubulin). Fold changes in protein expression were calculated relative to untreated control cells. Results are representative of three independent experiments. Statistical significance between treatment groups was determined using one-way ANOVA. Error bars represent SD. In all panels, *, *p* ≤ 0.05; **, *p* ≤ 0.01; ***, *p* ≤ 0.001; ****, *p* ≤ 0.0001

To evaluate the downstream effects of CDK4 inhibition on cell-cycle regulatory programmes, we examined the impact of AU2-94 on proliferation-associated proteins in LNCaP and PC3 cells, representing AR-driven prostate cancer and AR-null castration-resistant prostate cancer (CRPC), respectively. Consistent with the reduction in p-RB observed in Fig. 2A, immunoblotting revealed that AU2-94 reduced the expression of canonical E2F1 transcriptional targets, including CDK2, cyclin A2, cyclin B1, thymidine kinase 1 (TK1), and topoisomerase IIα, in a dose-dependent manner (Fig. 3A and B). AU2-94 also reduced FOXM1 expression in both LNCaP and PC3 cells.

Atirmociclib similarly suppressed these proliferation-associated markers in LNCaP and PC3 cells. However, at the highest concentration tested (4 µM), expression of several proteins partially recovered, which may reflect off-target engagement (Fig. 3A and B). Collectively, these findings support the conclusion that AU2-94 elicits the expected downstream consequences of CDK4 inhibition in both AR-driven and AR-null CRPC cells.

### AU2-94 exhibits potent antitumour efficacy in clinically relevant models of aggressive prostate cancer

**Fig. 4:**
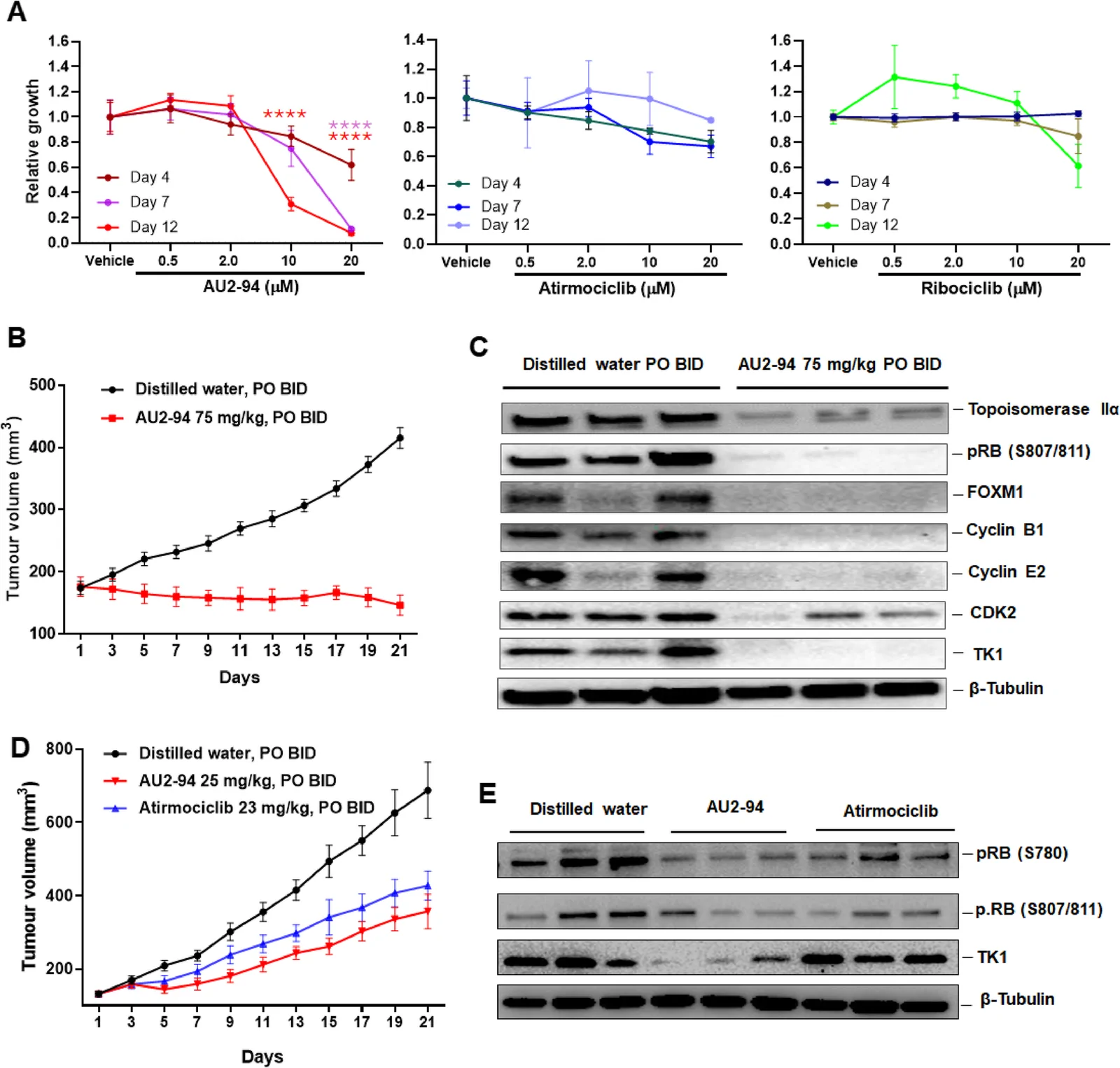
Efficacy of AU2-94 in diverse models of advanced prostate cancer.** A** Effects of AU2-94, atirmociclib, and ribociclib on 224R-Cx organoids. Organoid viability was determined using PrestoBlue viability assays at the indicated time points following treatment. Data represent the mean ± SEM of quintuplicate samples. Treatment groups were compared with vehicle controls using ANOVA followed by Dunnett’s multiple comparison tests. **B** LNCaP tumour-bearing mice (*n* = 5 per group) were treated with vehicle (distilled water PO BID) or AU2-94 (75 mg/kg PO BID) for 21 days. Tumour volume was measured throughout the treatment period. **C** LNCaP tumours were collected 12 h after the final dose on day 21, and protein lysates from three individual tumours per treatment group were analysed by western blotting. **D** PC3 tumour–bearing mice (*n* = 10 per group) were treated for 21 days with vehicle (distilled water, PO BID), AU2-94 (25 mg/kg, PO BID), or atirmociclib (23 mg/kg, PO BID). Tumour volume was measured throughout the treatment period. **E** PC3 tumours were collected 12 h after the final dose on day 21, and protein lysates from three individual tumours per treatment group were analysed by western blotting. Error bars indicate SEM. BID: twice-daily dosing. In **A**, ****p ≤ 0.0001

Encouraged by the activity and selectivity of AU2-94 in cell lines, we next evaluated its therapeutic potential in more clinically relevant model systems. First, we examined its activity in organoids established from the patient-derived xenograft (PDX) 224R-Cx, which has been characterised as AR-negative, RB-proficient, and positive for neuroendocrine markers [49]. AU2-94 reduced the growth of 224R-Cx organoids at concentrations ≥ 10 µM, whereas these organoids were largely non-responsive to atirmociclib or ribociclib (Fig. 4A).

We then assessed AU2-94 in vivo using an AR-driven LNCaP xenograft model. LNCaP-bearing mice were treated with vehicle (distilled water, PO BID) or AU2-94 (75 mg/kg, PO BID) for 21 days. AU2-94 significantly delayed tumour growth, achieving a treatment-to-control tumour volume ratio (T/C) of 35.22% at day 21 (Fig. 4B; Table 1). This growth suppression was evident as early as day 7 (T/C = 68.84%; *p* = 0.0038) and was sustained throughout the study period (Fig. 4B; Table 1). AU2-94 was well tolerated, with no overt toxicity, treatment-associated body weight loss, or adverse clinical signs observed, as assessed using a clinical record scoring system (Supplementary Methods). Statistical analyses of tumour volume and body weight changes are provided in Supplementary Tables S3 and S4. To confirm target engagement, immunoblotting of tumour lysates demonstrated reduced RB phosphorylation (p-S807/811) in AU2-94-treated tumours relative to controls. This was accompanied by downregulation of E2F1-regulated markers, including topoisomerase IIα, cyclin B1, CDK2, TK1 and FOXM1 (Fig. 4C and Supplementary Fig. S2A). Next, we compared AU2-94 and atirmociclib in the castration-resistant PC3 xenograft model. Given the broader CDK inhibition profile of atirmociclib (Fig. 2E), we selected mid-range doses to minimise potential confounding from off-target effects and to better attribute antitumour activity to CDK4 inhibition. Mice were treated for 21 days with AU2-94 (25 mg/kg, PO BID) or atirmociclib (23 mg/kg, PO BID) (A dose of 25 mg/kg AU2-94 (hydrochloride salt) is molar-equivalent to 23 mg/kg atirmociclib (free base)). Both agents significantly (*p* ≤ 0.0001) suppressed tumour growth (T/C = 51.95% for AU2-94 and, T/C = 62.19% for atirmociclib, on day 21; Fig. 4D; Table 1). Statistical analyses of tumour volume ratios and body weight changes are provided in Supplementary Tables S3 and S4. Consistent with on-target CDK4 inhibition, immunoblotting of tumour lysates revealed reduced RB phosphorylation (p-S780 and p-S807/811) and decreased TK1 expression following treatment, with AU2-94 producing a more pronounced inhibitory effect (Fig. 4E and Supplementary Fig. S2B).

Collectively, these findings demonstrate that selective CDK4 inhibition has activity across multiple clinically relevant prostate cancer contexts and support orally administered AU2-94 as a candidate treatment strategy for aggressive prostate cancer subtypes.

**Table 1:** Percent tumour volume ratio (T/C) over time in xenograft models

Model	Treatment Vs vehicle	Dose (mg/kg)	T/C % on Days										
			1	3	5	7	9	11	13	15	17	19	21
LNCaP	AU2-94	75	101.29	85.98	74.50	68.84	64.39	57.97	54.47	51.47	49.80	42.60	35.22
PC3	AU2-94	25	100	93.40	69.10	67.63	59.98	59.59	58.64	52.91	55.05	53.65	51.95
PC3	Atirmociclib	23	100	93.41	79.76	82.24	79.07	75.51	71.74	69.03	66.75	65.06	62.19

### CDK4 inhibition modulates AR-associated transcriptional programmes and can be effectively combined with AR-targeted therapy

**Fig. 5:**
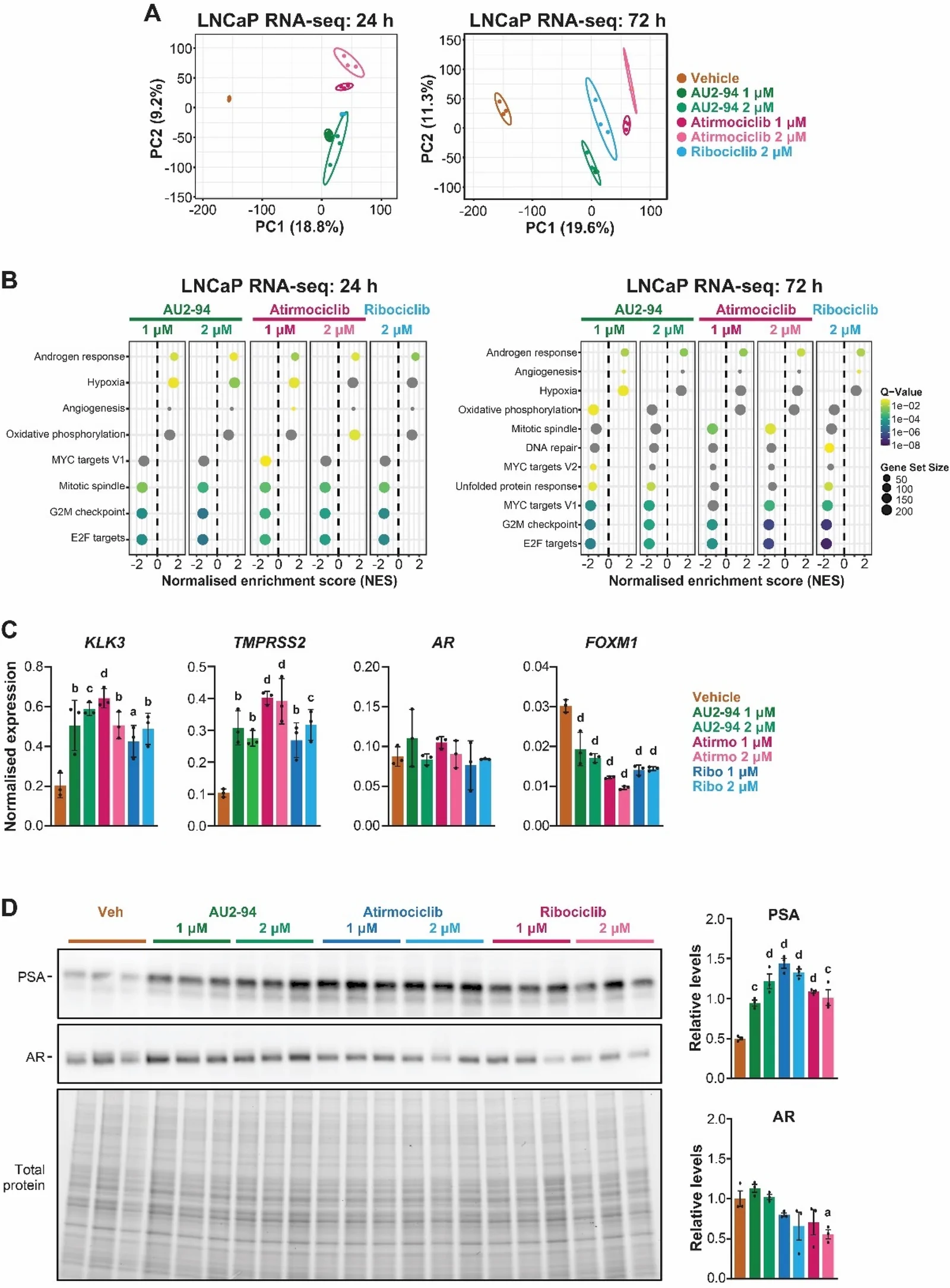
CDK4 inhibition activates AR signalling in prostate cancer.** A** Principal component analysis of gene expression from RNA-seq data following treatment of LNCaP cells with indicated drugs at 24 h (left) or 72 h (right). The plot was generated using ClustVis [59] after applying unit variance scaling to each element. **B** Hallmark gene sets affected by treatment with CDK4 inhibitors, as determined by gene set enrichment analysis. Gene sets significantly altered (q < 0.1) in at least one treatment are shown. NES, normalised enrichment score. **C** Effects of CDK4 inhibition on *KLK3* and *TMPRSS2* (AR target genes), *FOXM1* (an E2F target gene) and *AR* in LNCaP cells, evaluated by qRT-PCR. Error bars are ± SD. *GAPDH* and *ACTB* were used for normalisation. **D** Effects of CDK4 inhibition on AR and PSA (encoded by the *KLK3* gene) protein levels in LNCaP cells, evaluated by western blotting. The graphs on the right show relative levels of each protein normalised to total protein. Error bars are ± SEM. For panels C and D, statistical differences between groups were assessed using ordinary one-way ANOVA and Dunnett’s multiple comparisons testing (a, *p* ≤ 0.05; b, *p* ≤ 0.01; c, *p* ≤ 0.001; d, *p* ≤ 0.0001)

Our in vitro and in vivo studies demonstrated that CDK4 inhibition is efficacious across a broad spectrum of advanced prostate cancer models, including AR-driven and AR-independent subtypes. Nevertheless, most advanced and metastatic prostate tumours remain reliant on AR signalling, which continues to represent a central therapeutic target [6]. We therefore explored how CDK4 inhibition influences AR activity as a first step in evaluating the rationale for combining this approach with clinically approved AR-targeted agents. Transcriptomic profiling by RNA sequencing was undertaken in AR-driven LNCaP cells following treatment with AU2-94, atirmociclib, or ribociclib for 24–72 h. Each treatment induced widespread transcriptional changes, with hundreds of transcripts differentially expressed (adjusted *p* ≤ 0.05, log_2_ fold-change ≥ 1) (Supplementary Table S5 and Supplementary Dataset S1). Principal component analysis (PCA) demonstrated pronounced treatment-associated transcriptomic shifts and tight clustering of biological replicates (Fig. 5A).

Consistent with CDK4 pathway blockade, gene sets related to E2F activity and cell-cycle progression, including the hallmark “E2F targets”, “G2M checkpoint”, and “MYC targets” gene sets, were suppressed across all inhibitor treatments, most notably following 72 h of treatment (Fig. 5B). Interestingly, the Hallmark “Androgen response” gene set was consistently upregulated following CDK4 inhibition at both time points (Fig. 5B), and this effect was also observed for additional well-annotated gene sets used to measure AR activity (Supplementary Fig. S3A). Increased expression of prototypical AR target genes (*KLK3* and *TMPRSS2*), following CDK4 inhibition was validated by qRT-PCR (Fig. 5C). Moreover, CDK4 inhibitor-mediated induction of the protein product of *KLK3*, prostate-specific antigen (PSA), was confirmed by western blotting (Fig. 5D). Importantly, elevated levels of AR targets in response to CDK4 inhibition were not accompanied by increased mRNA or protein abundance of AR itself (Fig. 5C-D), suggesting that this phenomenon occurred via modulation of AR activity rather than its expression. Supporting this concept, CDK4 inhibitor-mediated induction of *KLK3*/PSA was reversed by treatment with the AR inhibitor, enzalutamide (Supplementary Fig. S3B-C).

**Fig. 6:**
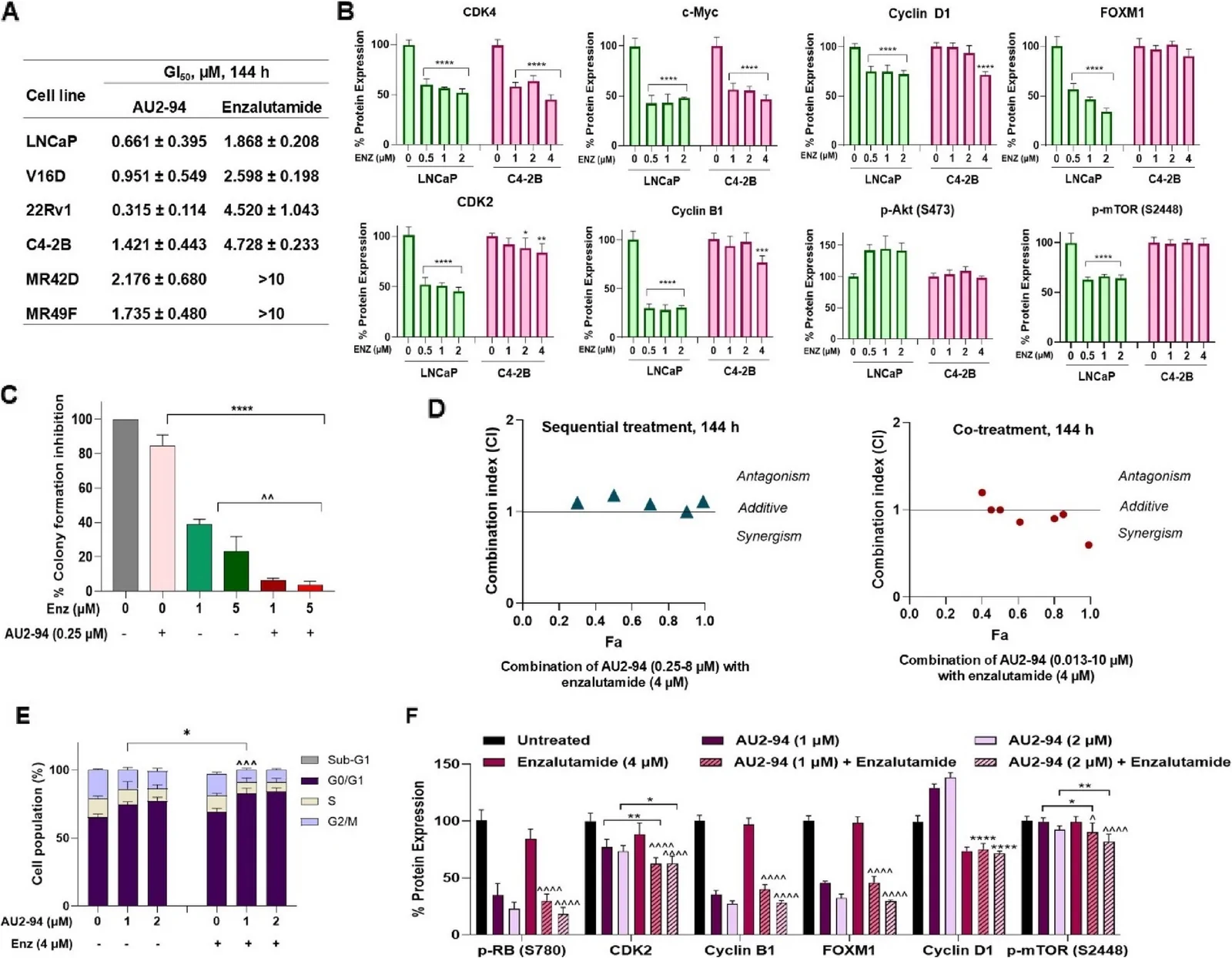
Combinatorial effects of AU2-94 and enzalutamide in CRPC cells. **A** A panel of prostate cancer cell lines was treated with enzalutamide or AU2-94 for 144 h. GI_50_ values represent the mean ± SD from at least three independent experiments. **B** Changes in protein expression were assessed in LNCaP and C4-2B cells following 24 h of treatment with enzalutamide concentrations selected according to their respective GI₅₀ values. Statistical significance difference between groups was assessed using ordinary one-way ANOVA. Error bars indicate SD. *, *p* ≤ 0.05; **, *p* ≤ 0.01; ***, *p* ≤ 0.001; ****, *p* ≤ 0.0001 compared with untreated cells. **C** Combination effects of AU2-94 and enzalutamide in C4-2B cells were determined by colony formation assays. Cells were treated with AU2-94 (0.25 µM), enzalutamide (1 and 5 µM), or the combination for 10 days. **D** Drug interactions following sequential or concurrent treatment were assessed by calculating combination indices using CompuSyn software. For sequential treatment, AU2-94 was tested across five concentrations (0.25–8 µM) using a 1:2 serial dilution series. For concurrent treatment, AU2-94 was tested across seven concentrations (0.013–10 µM) using a 1:3 serial dilution series. **E** Cell-cycle distribution was analysed by PI staining following 24 h of treatment. **F** Protein expression levels were assessed after 24 h of treatment using the AlphaLISA^®^ SureFire^®^ Ultra™ assay. Data shown are representative of three independent experiments. Statistical significance difference between groups was assessed using ordinary one-way ANOVA. Error bars indicate SD. *, *p* ≤ 0.05; **, *p* ≤ 0.01; ****, *p* ≤ 0.0001 compared with AU2-94-treated cells. ^, *p* ≤ 0.05; ^^, *p* ≤ 0.01; ^^^, *p* ≤ 0.001, ^^^^, *p* ≤ 0.0001 compared with enzalutamide-treated cells

Having evaluated the effects of CDK4 inhibition on AR signalling, we next asked whether reciprocal regulation occurs; specifically, whether AR blockade with enzalutamide alters the abundance and/or activity of CDK4 and related cell-cycle regulators. Enzalutamide is a non-steroidal AR antagonist widely used in metastatic prostate cancer and has demonstrated clinical benefit in both castration-sensitive and castration-resistant disease [60, 61]. To identify appropriate models, we first profiled enzalutamide sensitivity across a panel of prostate cancer cell lines. Three tiers of responsiveness were observed: LNCaP and V16D cells were the most sensitive; 22Rv1 and C4-2B cells displayed intermediate sensitivity; and MR42D and MR49F cells were the least sensitive, with GI₅₀ values > 10 µM (Fig. 6A). Based on their shared lineage but divergent enzalutamide responses, LNCaP and C4-2B cells were selected to evaluate the impact of AR antagonism on CDK4 and cell-cycle effectors. Cells were treated with enzalutamide at one-quarter, one-half, and one-fold GI₅₀ concentrations to account for differential drug sensitivity. Enzalutamide significantly reduced CDK4 and c-Myc expression in both cell lines. In LNCaP cells, enzalutamide additionally reduced CDK2, cyclin B1, cyclin D1, FOXM1, and p-mTOR (Fig. 6B**)**.

Since CDK4 inhibition was associated with enrichment of AR-associated transcriptional programmes, and enzalutamide suppressed CDK4 and other cell-cycle regulators, we hypothesised that combined CDK4 and AR inhibition may provide enhanced antitumour activity. Consistent with this, co-treatment of AU2-94 (0.25 µM) and enzalutamide (1 and 5 µM) in C4-2B cells suppressed clonogenic growth more effectively than either agent alone (Fig. 6C). Combination index (CI) analysis indicated an additive interaction (Fig. 6D). A sequential treatment schedule in which AU2-94 was administered 72 h prior to enzalutamide also produced an additive interaction (Fig. 6D). Given the consistent additive effects observed with concurrent administration, subsequent experiments were performed using the co-treatment schedule.

We next examined the effects of combination treatment on cell-cycle distribution and proliferation-related proteins. Co-treatment with AU2-94 (1 µM) and enzalutamide (4 µM) increased the proportion of cells in G0/G1 phase (Fig. 6E). Relative to enzalutamide alone, the combination further reduced p-RB (S780), CDK2, cyclin B1 and p-mTOR (S2448) in C4-2B cells **(**Fig. 6F). However, compared with AU2-94 monotherapy, the combination did not produce substantial additional suppression of these CDK4 signalling outputs, suggesting that the enhanced growth inhibition may reflect additional downstream effects of AR blockade beyond the canonical CDK4–RB–E2F axis.

### Combining AU2-94 and alpelisib produces synergistic antiproliferative effects in enzalutamide-resistant prostate cancer

**Fig. 7:**
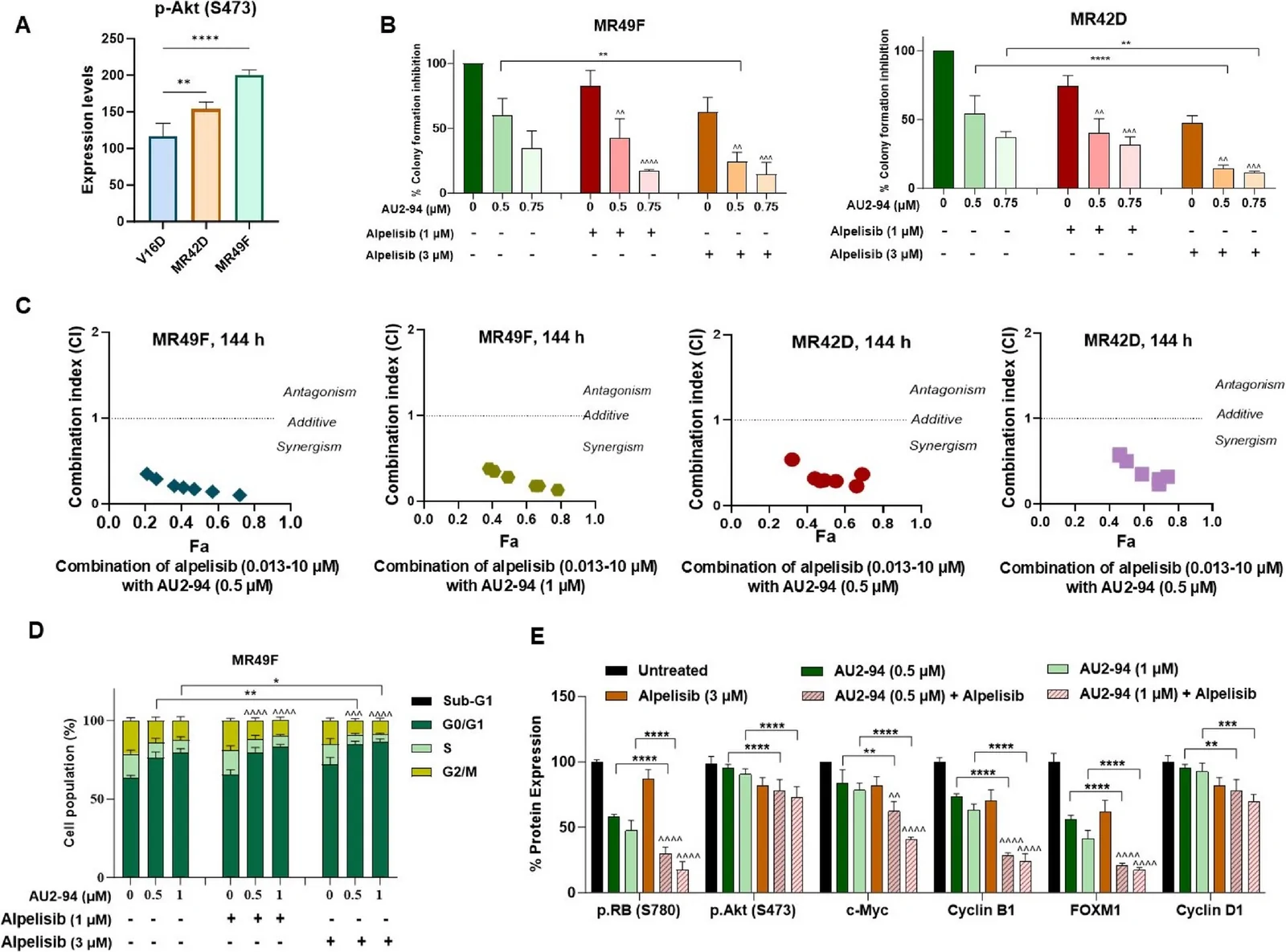
Combination effects of AU2-94 and alpelisib in enzalutamide-resistant MR49F and MR42D cells. **A** Compared with parental V16D cells, enzalutamide-resistant MR42D and MR49F cells showed higher expression levels of p-Akt (S473). **B** MR49F and MR42D cells were treated with AU2-94 (0.5 and 0,75 µM), alpelisib (1 and 3 µM), or the combination for 10 days. Colonies were stained, imaged, and quantified using ImageJ software. **C** Drug interactions following concurrent treatment were assessed by calculating the CI using CompuSyn software. Alpelisib was tested across seven concentrations (0.013–10 µM) using a 1:3 serial dilution series. **D** Cell-cycle distribution was analysed by PI staining following 24 h treatment. (**E**) Protein expression levels were assessed after 24 h treatment using the AlphaLISA^®^ SureFire^®^ Ultra™ assay. Data shown are representative of three independent experiments. Statistical significance between groups was assessed using ordinary one-way ANOVA. Error bars indicate SD. *, *p* ≤ 0.05; **, *p* ≤ 0.01; ***, *p* ≤ 0.001; ****, *p* ≤ 0.0001 compared with single agent of AU2-94. ^, *p* ≤ 0.05; ^^, *p* ≤ 0.01; ^^^, *p* ≤ 0.001; ^^^^, *p* ≤ 0.0001 compared with single agent of alpelisib

Hyperactivation of the PI3K/Akt pathway is a well-established mechanism of resistance to AR-targeted therapies in the CRPC setting [62, 63]. Consistent with this, we observed significantly higher levels of phosphorylated Akt (S473) in enzalutamide-resistant MR42D and MR49F cells than in the parental V16D cells (Fig. 7A). On this basis, we examined the therapeutic potential of co-targeting CDK4 and PI3K using AU2-94 and alpelisib, a PI3Kα inhibitor, in enzalutamide-resistant cells.

Cell viability assays showed that both agents reduced proliferation in MR42D and MR49F cells. AU2-94 exhibited GI₅₀ values of 2.18 µM in MR42D cells and 1.73 µM in MR49F cells, whereas alpelisib showed GI₅₀ values of 9.01 µM and 10.13 µM, respectively. Co-treatment with AU2-94 and alpelisib resulted in greater growth inhibition than either monotherapy in both models (Fig. 7B). CI analyses demonstrated synergistic interactions, with the effect being more pronounced in MR49F cells (Fig. 7C). Consistent with this interaction, co-treatment of MR49F cells with AU2-94 (0.5 µM or 1 µM) and alpelisib (1 µM or 3 µM) increased G0/G1 cell accumulation relative to either agent alone, particularly at the higher alpelisib concentration (Fig. 7D). At the molecular level, combination treatment was associated with greater reductions in p-RB (S780), c-Myc, cyclin B1, and FOXM1 relative to either monotherapy (Fig. 7E).

Together, these findings demonstrate that concurrent inhibition of CDK4 and PI3Kα produces a synergistic antiproliferative effect in enzalutamide-resistant prostate cancer cells. This interaction was associated with enhanced G0/G1 accumulation and greater suppression of proliferation-related markers, suggesting that co-targeting these pathways may limit compensatory signalling in resistant disease states.

### AU2-94 synergises with docetaxel against AR-independent CRPC

Docetaxel is a taxane chemotherapeutic agent used in advanced prostate cancer; however, its efficacy is often limited in the CRPC setting [64]. Combination strategies have been explored to extend the therapeutic benefit of docetaxel, but clinical translation has largely been constrained by dose-limiting toxicities [65, 66]. Based on prior evidence suggesting that suppression of E2F1-driven transcription and restriction of cell-cycle re-entry from quiescent (G0 phase) states can enhance taxane efficacy [67, 68], we postulated that CDK4 inhibition may sensitise prostate cancer cells to docetaxel.

**Fig. 8:**
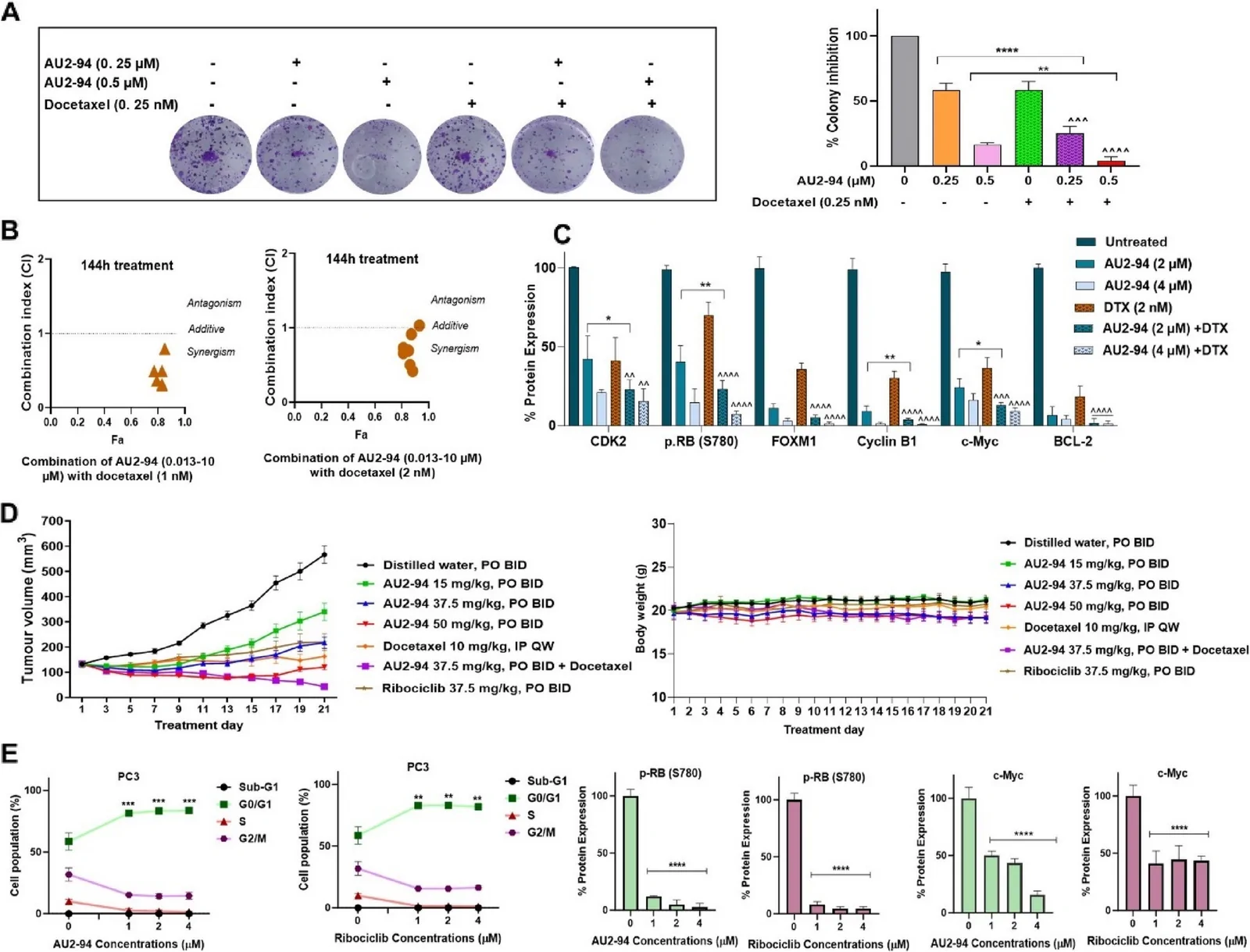
Effects of AU2-94 and docetaxel against PC3 cells and xenograft models. **A** PC3 cells were treated with AU2-94 and docetaxel, either as single agents or in combination, for 10 days. Clonogenic growth was assessed using colony formation assays and quantified using ImageJ software. **B** Combination indices between AU2-94 and docetaxel upon 144 h of treatment were determined using CompuSyn software. AU2-94 was tested across seven concentrations (0.013–10 µM) using a 1:3 serial dilution series. **C** Alterations in protein expression following co-treatment were determined using the AlphaLISA^®^ SureFire^®^ Ultra™ assay. Data are representative of three independent experiments. Statistical significance between groups was assessed using one-way ANOVA. Error bars indicate SD. *, *p* ≤ 0.05; **, *p* ≤ 0.01; ****, *p* ≤ 0.0001 compared with AU2-94-treated cells. ^^, *p* ≤ 0.01; ^^^, *p* ≤ 0.001; ^^^^, *p* ≤ 0.0001 compared with docetaxel-treated cells. **D** Efficacy of AU2-94, docetaxel, and their combination in the PC3 xenograft model. Tumour volume and body weight changes were measured throughout the 21-day treatment period. Error bars indicate SEM. **E** Comparison of the effects of AU2-94 and ribociclib in PC3 cells in vitro. Data shown represent the mean of three independent experiments. Statistical significance between groups was assessed using ordinary one-way ANOVA. Error bars indicate SD. **, *p* ≤ 0.01; ***, *p* ≤ 0.001; ****, *p* ≤ 0.0001 compared with control. BID: twice-daily dosing; QW: once-weekly dosing

Consistent with this hypothesis, concurrent treatment of AR-independent PC3 cells with AU2-94 and docetaxel resulted in greater growth inhibition than either agent alone, as demonstrated by a marked reduction in clonogenic capacity (Fig. 8A). CI analysis confirmed a predominantly synergistic interaction between AU2-94 and docetaxel (Fig. 8B). At the molecular level, combination treatment more strongly suppressed p-RB (S780), CDK2, c-Myc, and cyclin B1 than either monotherapy (Fig. 8C). Collectively, these findings indicate that CDK4 inhibition enhances docetaxel activity, which was associated with greater suppression of RB/E2F1 signalling. To investigate whether this interaction translates in vivo, male BALB/c nude mice bearing established PC3 tumours were treated for 21 days with: (i) vehicle, (ii–iv) AU2-94 (15, 37.5, or 50 mg/kg, PO BID), (v) docetaxel (10 mg/kg, IP once weekly), (vi) AU2-94 (37.5 mg/kg, PO BID) plus docetaxel (10 mg/kg, IP once weekly), or (vii) ribociclib (37.5 mg/kg, PO BID). The docetaxel regimen was selected based on clinically relevant human-equivalent dosing schedules of 75–100 mg/m² every three weeks [11]. AU2-94 monotherapy inhibited tumour growth in a dose-dependent manner, resulting in T/C values of 60%, 38%, and 21% at day 21 for 15, 37.5, and 50 mg/kg, respectively (Fig. 8D; Table 2). Notably, co-administration of AU2-94 with docetaxel further enhanced antitumour activity (Fig. 8D). This combination reduced tumour volume to a T/C of 8% at day 21, which was significantly lower than that achieved by docetaxel (T/C = 29%) or AU2-94 (T/C = 38%) monotherapy (Fig. 8D; Table 2). All treatment regimens, including the combination, were generally well tolerated, with no treatment-associated body weight loss or adverse clinical signs, as assessed using a clinical record scoring (CRS) system (Fig. 8D, Supplementary Methods). Statistical analyses of tumour volume ratios and body weight changes are presented in Supplementary Tables S3 and S4.

**Table 2:** Percent tumour volume ratio (T/C) over time in PC3 xenograft model

Treatment groups	T/C % on Days										
	1	3	5	7	9	11	13	15	17	19	21
Vehicle vs. AU2-94 15 mg/kg, PO BID	99.92	79.91	71.56	66.18	61.28	56.93	58.85	58.85	58.23	60.76	60.05
Vehicle vs. AU2-94 37.5 mg/kg, PO BID	101.21	75.45	63.75	58.62	54.69	47.28	41.76	42.30	37.46	40.82	38.40
Vehicle vs. AU2-94 50 mg/kg, PO BID	100.45	66.41	51.63	47.96	40.44	28.04	23.68	23.47	19.18	22.29	21.26
Vehicle vs. Docetaxel 10 mg/kg, IP QW	100.98	77.44	73.25	74.99	69.45	50.93	43.70	40.05	35.65	29.51	28.84
Vehicle vs. AU2-94 37.5 mg/kg + Docetaxel 10 mg/kg	100.83	66.79	58.33	52.96	47.12	33.45	25.31	21.47	14.85	12.45	7.69
Vehicle vs. Ribociclib 37.5 mg/kg, PO BID	97.74	76.78	76.53	75.68	73.82	57.74	52.28	49.36	43.90	43.52	38.80

Because PC3 cells express CDK6, we also compared selective CDK4 inhibition with dual CDK4/6 blockade by evaluating ribociclib in the same experiment. Ribociclib (37.5 mg/kg, PO BID) reduced tumour growth to a T/C of 39%, comparable to AU2-94 at the same dose (Fig. 8D; Table 2). Concordantly, in vitro analyses showed no significant difference between AU2-94 and ribociclib in inducing G0/G1 arrest or suppressing p-RB (S780) and c-Myc in PC3 cells (Fig. 8E), suggesting comparable functional effects in this model.

Together, these findings support AU2-94 as a promising therapeutic candidate for AR-independent CRPC, with activity as a single agent and in combination with docetaxel.

## Discussion

Dysregulation of the cyclin D–CDK4/6–RB axis represents a central proliferative engine in prostate cancer across both AR-dependent and AR-independent disease states [14]. However, CDK4 and CDK6 do not contribute equivalently to prostate cancer growth. Using large-scale transcriptomic and functional genomic datasets, we found that prostate cancer exhibits a stronger dependency on CDK4 than on CDK6. This differential reliance likely reflects both quantitative differences in expression and qualitative divergence in biological function. While CDK4 primarily drives canonical RB phosphorylation and G1/S cell-cycle progression, CDK6 has been implicated in context-dependent regulation of luminal differentiation states and AR transcriptional programmes [69]. Consistent with this, our analyses also demonstrated that elevated CDK6 expression was associated with a more favourable clinical course relative to CDK4, supporting the notion that CDK6 may serve as a marker of an AR-responsive phenotype rather than a dominant proliferative driver.

The concept that proliferative dependency in prostate cancer is preferentially mediated through CDK4-mediated RB control provides a rationale for selective CDK4 inhibition as a strategy to preserve antitumour efficacy while potentially reducing haematologic toxicity associated with CDK6 inhibition [56]. Consistent with this molecular framework, AU2-94 elicited antiproliferative effects comparable to those of dual CDK4/6 inhibition across multiple aggressive prostate cancer models, including a patient-derived neuroendocrine prostate cancer organoid model and in vivo tumour xenografts representing both androgen receptor–dependent and –independent disease states. AU2-94 also retained robust in vivo antitumour activity comparable to ribociclib in the PC3 xenografts, suggesting that selective CDK4 inhibition can remain effective even in tumours with substantial CDK6 expression. Mechanistically, AU2-94 suppressed RB/E2F1 output, reduced cell-cycle and G2/M checkpoint programmes, and downregulated key proliferation-associated factors.

Beyond its canonical role in cell-cycle regulation, CDK4 inhibition was associated with enrichment of AR-response signatures, suggesting broader effects on transcriptional state in prostate cancer cells. Prostate cancer progression is characterized by dynamic interplay between proliferative drivers and lineage-defining transcriptional networks, with resistance frequently emerging through alteration and/or attenuation of canonical AR signalling coupled with induction of compensatory pathways [5]. Clinically, tumours with enriched AR signalling derive greater benefit from anti-androgen therapies, including higher rates of pathological response [70]. Importantly, we observed elevated expression of androgen-regulated genes following CDK4 inhibition, suggesting that suppression of E2F-driven proliferation is associated with increased AR transcriptional activity. Such transcriptional changes may reinforce lineage dependency and enhance susceptibility to pharmacological AR blockade, potentially contributing to the predominantly additive effects observed with AU2-94 and enzalutamide. Similar phenomena have been reported in oestrogen receptor-positive breast cancer, where CDK4/6 inhibition drives RB-dependent transcriptional reprogramming and activates oestrogen-regulated enhancers linked to endocrine sensitivity [71, 72]. Together, these observations suggest that CDK4 inhibition may be associated with broader transcriptional state changes in hormone-driven malignancies, although the precise mechanisms underlying these effects require further investigation.

In addition to this transcriptional reprogramming, selective CDK4 inhibition may target a downstream node within adaptive resistance pathways. In the context of resistance to AR-targeted therapies such as enzalutamide, tumour cells frequently engage compensatory survival signalling, most notably the PI3K/Akt pathway, to sustain proliferative capacity [73]. Although PI3K inhibitors have been proposed as a strategy to overcome this resistance mechanism, their clinical efficacy has been limited by adaptive feedback that restores proliferative output, including reactivation of cyclin D1–CDK4 signalling [74–78]. Consistent with this model, combined inhibition of CDK4 and PI3K signalling with AU2-94 and alpelisib produced synergistic antiproliferative effects in enzalutamide-resistant cells, accompanied by suppression of proliferation-associated markers. These findings suggest that co-targeting PI3K signalling and CDK4 may limit compensatory cell-cycle re-entry in therapy-resistant disease states.

Another major clinical challenge in prostate cancer management is AR-negative CRPC, which is intrinsically resistant to AR-targeted therapies and for which effective treatment options remain limited, making docetaxel-based chemotherapy as an important treatment approach [11]. We observed that CDK4 inhibition enhanced the cytotoxic efficacy of docetaxel in AR-independent cancer cells. The enhanced antitumour activity observed with AU2-94 and docetaxel may be associated with combined effects on RB–E2F signalling and cell-cycle progression. It has been shown that modulation of E2F1 and FOXM1 expression may disturb proliferation-associated pathways, resulting in increased sensitivity to taxane treatment [79]. Accordingly, combined CDK4 inhibition and taxane exposure appeared to produce greater suppression of proliferative signalling and tumour cell growth; however, the extent to which these changes contribute to the enhanced response to docetaxel remains to be determined.

Synergy between CDK4/6 inhibitors and taxanes has also been previously demonstrated in prostate cancer; however, clinical translation has been constrained by overlapping haematologic toxicities. In metastatic CRPC, the combination of ribociclib and docetaxel, despite demonstrating antitumour activity, was associated with high rates of grade III/IV neutropenia, necessitating dose modifications and limiting therapeutic feasibility [23]. In our in vivo study, AU2-94 combined with docetaxel enhanced antitumour efficacy without overt signs of toxicity. Tolerability was supported by maintenance of animal body weight and a comprehensive clinical scoring assessments that evaluated parameters such as hydration, respiratory effort, and activity levels, none of which indicated treatment-related distress. These findings are consistent with the proposed therapeutic advantage of CDK4-selective inhibitors [30, 31], which is biologically grounded in the distinct roles of CDK4 and CDK6 within the haematopoietic compartment [25, 26]. In line with this concept, our previous study directly comparing AU2-94 and dual CDK4/6 inhibitors demonstrated that AU2-94 exerted a milder antiproliferative effect on haematopoietic stem and progenitor cells, preserved the population of Ki67-positive cells within bone marrow, and maintained neutrophil and lymphocyte counts that were otherwise significantly depleted following dual CDK4/6 inhibition at equivalent doses [27]. Together, these findings support the hypothesis that selective CDK4 inhibition may reduce burden on haematopoietic turnover relative to dual CDK4/6 blockade; however, this remains to be validated in the context of prostate cancer therapy.

Despite growing clinical momentum for CDK4-selective strategies, comparative biochemical and cellular characterisation of agents within this emerging class remains limited and, in some instances, discordant. Given that even subtle differences in kinase selectivity can influence cell-cycle dynamics, transcriptional adaptation, and tumour dependency states, defining the kinase inhibition spectrum of individual compounds is likely to be important for biomarker-driven patient stratification and therapeutic positioning. Our biochemical analyses demonstrated that AU2-94 and atirmociclib exhibit comparable nanomolar potency against CDK4. This finding contrasts with previous reports suggesting that AU2-94 (CDDD2-94) lacks CDK4 selectivity [30]. However, details regarding the chemical characteristics of the compound material used in that study were not disclosed; therefore, differences in compound sourcing, chemical structure, purity, and assay configuration may explain the inter-study variability. Notably, our current findings are concordant with previous kinome-wide profiling that demonstrated marked selectivity of AU2-94 for CDK4 over other CDK family members and kinases [27]. In contrast, in the present study, atirmociclib displayed potent activity against other CDK family members, including CDK1, CDK2, CDK7, and CDK9. Engagement of these non-CDK4 targets was associated with altered cell-cycle distribution, transcriptional reprograming, and tumour dependency states, consistent with the observed G2/M arrest phenotype and activity in RB-deficient contexts. By contrast, the greater CDK4 selectivity of AU2-94 supports a more focused suppression of RB-driven proliferative circuitry. Collectively, these results indicate that selective CDK4 inhibition and broader CDK targeting represent mechanistically distinct pharmacologic strategies that may confer different therapeutic profiles depending on tumour context.

In summary, our findings identify AU2-94 as a promising therapeutic candidate in prostate cancer and support the concept that selective CDK4 inhibition can effectively suppress E2F-driven proliferative circuitry. While this study and prior evidence [30] implicate CDK4 as a dominant proliferative driver in prostate cancer, the potential for functional compensation by CDK6 cannot be excluded and warrants further mechanistic investigation. In addition, although combination strategies demonstrated enhanced anti-tumour activity, the mechanisms driving these interactions require further investigation to identify the molecular mediators underlying these effects. Such studies may refine biomarker strategies, clarify which tumour contexts are optimally suited to CDK4 inhibition versus broader CDK targeting, and inform the rational development of combination therapies for the clinic.

## Supplementary Information

Supplementary Material 1.

Supplementary Material 2.

## Data Availability

The RNA-sequencing data are deposited into the Gene Expression Omnibus database under accession number GSE330358 and are available at the following URL: https://www.ncbi.nlm.nih.gov/geo/query/acc.cgi? acc=GSE330358.

## Abbreviations

ADP: Adenosine diphosphate
AR: Androgen receptor
ATP: Adenosine triphosphate
BID: Bis in die (twice daily)
CDK: Cyclin-dependent kinase
CI: Combination index
CRPC: Castration-resistant prostate cancer
DMSO: Dimethyl sulfoxide
DTX: Docetaxel
E2F1: E2F transcription factor 1
ENZ: Enzalutamide
FBS: Foetal bovine serum
FCS: Foetal calf serum
FOXM1: Forkhead box M1
GI_50_: Half-maximal growth inhibitory concentration
GSEA: Gene set enrichment analysis
IC_50_: Half-maximal inhibitory concentration
IP: Intraperitoneal
KLK3: Kallikrein-related peptidase 3
mTOR: Mechanistic target of rapamycin
PBS: Phosphate-buffered saline
PCA: Principal component analysis
PDX: Patient-derived xenograft
PI: Propidium iodide
PI3K: Phosphoinositide 3-kinase
PO: Per os (oral administration)
PSA: Prostate-specific antigen
QW: Quaque week (once weekly)
RB: Retinoblastoma protein
RNAi: RNA interference
SAGC: South Australian Genomics Centre
SEM: Standard error of the mean
SD: Standard deviation
TCGA: The Cancer Genome Atlas
TK1: Thymidine kinase 1
