# Supplementary material for: CDK4-selective inhibitor AU2-94 for the treatment of advanced and therapy-resistant prostate cancer
Source: J Exp Clin Cancer Res. 2026 Jun 17;45:188. doi: 10.1186/s13046-026-03763-x (PMC13520305; doi:10.1186/s13046-026-03763-x)
Supplement: Supplementary file 1 [file 13046_2026_3763_MOESM2_ESM.docx]

**Supplementary Methods**

**CDK4-selective inhibitor AU2-94 for the treatment of advanced and therapy-resistant prostate cancer**

Ava Safaroghli-azar^1^, Travis Croft^2^, Laychiluh Bantie Mekonnen^1^, Jimma Lenjisa^1^, Maja Dickel^2^, Raj K. Shrestha^2^, Samyukta Sita^3^, Nicholas Choo^3^, Michael Roach^2^, Sunita K.C. Basnet^1^, Mitchell G. Lawrence^3,4,5,6^, Luke A. Selth^2,7^*, Shudong Wang^1^*

^1^Drug Discovery and Development, School of Pharmacy and Biomedical Sciences, College of Health, Adelaide University, SA 5001, Australia.

^2^Flinders University, College of Medicine and Public Health, Flinders Health and Medical Research Institute, Bedford Park, SA 5042, Australia.

^3^Melbourne Urological Research Alliance, Biomedicine Discovery Institute, Monash University, Clayton, Victoria, 3800, Australia.

^4^Peter MacCallum Cancer Centre, Melbourne, Victoria, 3000, Australia.

^5^Sir Peter MacCallum Department of Oncology, The University of Melbourne, Melbourne, Victoria, 3010, Australia.

^6^Cabrini Institute, Cabrini Health, Malvern, Victoria, 3144, Australia.

^7^College of Health, Adelaide University, SA 5001, Australia.

*Correspondence: Shudong Wang, Email: [shudong.wang@adelaide.edu.au](mailto:shudong.wang@adelaide.edu.au)

Luke A. Selth, Email: [luke.selth@flinders.edu.au](mailto:luke.selth@flinders.edu.au)

**S1. Composition of CDK/Cyclin Kinase Reaction Master Mixes**

Kinase reactions were performed using recombinant CDK/cyclin complexes under ATP concentrations corresponding to the respective Km values for each kinase. All reactions were performed in 4× Kinase Reaction Buffer (4× KRB), comprising 160 mM Tris-HCl (pH 7.5), 80 mM MgCl₂, 0.5 mg/mL BSA, and 200 µM DTT. The buffer was diluted to 1× in the final reaction mixture. Reaction volumes were 4 µL unless otherwise stated.

**CDK1/cyclin B1**

Reactions contained recombinant CDK1/cyclin B1 (Thermo Fisher Scientific, Cat. #PV3292), histone H1 peptide (Mimotopes, Cat. #86708; sequence H-GGGPATPKKAKKL-OH), and ATP (Promega, Cat. #V9102). Final concentrations were 1 nM CDK1/cyclin B1, 10 µM histone H1 peptide, and 35 µM ATP.

**CDK7/cyclin H/MAT1**

Reactions contained CDK7/cyclin H/MAT1 (ProQinase, Cat. #0366-0360-4), CDK7/9tide (Mimotopes; sequence H-YSPTSPSYSPTSPSYSPTSPSKKKK-OH), and ATP (Promega, Cat. #V9102). Final concentrations were 25 nM CDK7/cyclin H/MAT1, 250 µM CDK7/9tide, and 4 µM ATP.

**CDK4/cyclin D1**

Reactions contained CDK4/cyclin D1 (Thermo Fisher Scientific, Cat. #PV4400), RBING peptide (Mimotopes; sequence H-INGSPRTPRRGQNR-OH), and ATP. Final concentrations were 40 nM CDK4/cyclin D1, 2 mM RBING peptide, and 150 µM ATP.

**CDK6/cyclin D3**

Reactions contained CDK6/cyclin D3 (SignalChem, Cat. #V6282), histone H1 peptide (Mimotopes, Cat. #86708), and ATP. Final concentrations were 40 nM CDK6/cyclin D3, 250 µM histone H1 peptide, and 200 µM ATP.

**CDK9/cyclin T1**

Reactions contained CDK9/cyclin T1 (ProQinase, Cat. #0371-0345-1), CDK7/9tide (Mimotopes; sequence H-YSPTSPSYSPTSPSYSPTSPSKKKK-OH), and ATP (Promega, Cat. #V9102). Final concentrations were 55 nM CDK9/cyclin T1, 250 µM CDK7/9tide, and 4 µM ATP.

**S2. Clinical Record Scoring (CRS) for *In Vivo* Toxicity Monitoring**

Animal Ethics Committee - Clinical Record Coversheet

| CRS | Clinical Criteria | Normal | Moderate abnormality | Significant/sustained abnormality |
| --- | --- | --- | --- | --- |
|  | **Score** | **0 (leave blank)** | **1** | **2** |
| **1** | Lack of, or abnormal movement | Active spontaneous free movement | Lack of spontaneous movement/hyperactivity | Reduced movement on stimulation/ excessive activity, frantic behaviour |
| **2** | Hunching | Extended back, normal movement and gait | Slight arching on movement | Arched on movement |
| **3** | Ruffled coat | Smooth sleek coat | Slight ruffling | Significant ruffling over entire coat |
| **4** | Abnormal breathing | Normal breathing in character and frequency | Increased respiratory rate or slight increased effort | Marked increase in respiratory rate with marked effort |
| **5** | Skin crinkling/tenting/sunken eyes | Soft, pliable, elastic, bright eyes (> 75% open) | Skin crinkled, slight tenting when skin lifted | Skin tenting and sunken eyes |
| **6** | Profuse/bloody diarrhoea | Well-formed stools | Profuse with cage smeared with diarrhoea | Bloody & profuse diarrhoea |
| **7** | Seizure/erratic movement | No evidence of seizure/erratic movement | Mild tremor/slight erratic movement | Prolonged whole-body seizure/continuous erratic movement |

**MONITORING AND WEIGHING FREQUENCY**

Individual mouse will be closely monitored daily for clinical signs of toxicity using this CRS.

Mice are to be weighed three times weekly; if weight loss is ≥ 10% weighing frequency will be increased to daily.

Calliper measurements for monitoring tumour growth three time weekly after commencing the treatment. Measuring frequency may be increased as agreed by the Uni Vets once the tumour volume is ≥1500 mm^3^.

**ACTIONS AND INTERVENTIONS**

| **TOTAL Score** | **Assessment** | **Actions/Interventions** |
| --- | --- | --- |
| **0** | Animal within normal limits | No interventions required. |
| **1** | Animal demonstrates slight abnormality | Monitor closely until resolved |
| **2** | Animal demonstrates moderate abnormality | Score 2 for any one criterion – consult Uni Vet  Cumulated 2: Increase monitoring to twice daily |
| **3** | Animal demonstrates moderate/sustained abnormality in multiple criteria | Consult University Veterinarian, follow advice  Increase monitoring to three times daily |
| **4 or greater** | Animal demonstrates significant or sustained abnormality | Immediate humanely kill |
| **Absolute humane endpoint criteria:**  **Laboured breathing**  **BCS < 2/5**  **Seizure/erratic movement** | Animal exhibits respiratory distress – gasping.  BSC < 2/5 signifies sharp vertebral processes, body is emaciated  Prolonged whole-body seizure/continuous erratic movement | Immediate humanely kill |
| **Tumour Volume** | Tumour volume ≥1500mm^3^ | Notify Uni Vets and increase measuring frequency as agreed by the Uni Vets |
|  | Tumour volume ≥ 2000mm^3^ | Immediately humanely kill |
| **Tumour ulceration** | Wet tumour surface or bleeding evident which does not dry or scab within 6 hours or overnight/ accompanying signs of ulcer inflammation/ persistent scratching or cleaning by the mouse | Consult University Veterinarian, follow advice  Increase monitoring to twice daily. In the absence of veterinary advice within 12 hours of contact, the animal will be humanely killed  Complete a Running Mortality Sheet. |
| **Weight loss %** | **Weight loss ≥ 10%** | Increase weighing frequency, give soaked food. If >10% weight loss persists for 48 hours, consult Uni Vet and follow advice or humanely kill. |
|  | **Weight loss ≥15%** | Humane endpoint reached; immediately humanely kill. |
| Note: Weight loss in growing animals is calculated against pre-interventional weight, control animals or standardized growth charts. If growing animals fall below the bottom of the growth curve for the background strain as provided from the supplier, consult the University Veterinarian or humanely kill.  If any neurological signs are observed, the University Veterinarian will be consulted. | | |

**Explanations**

a. Each animal is examined at each nominated monitoring time point.

- Each criterion is scored and the total score achieved by each animal must be recorded in the total score column. Training by the University Veterinarian or senior facility technician is required to ensure all personnel are consistent in terms of scoring.
- Scores are added and the score is recorded on the Monitoring Sheet. Each day of monitoring must be initialled by the person monitoring

b. Appropriate to the score, specific actions/interventions are undertaken.
c. Comments concerning abnormalities are recorded in the “Comments” section.

- Any abnormality that is observed to be of greater severity than the descriptions provided above, or a major deviation from normal beyond that approved, requires immediate consultation with the University Veterinarian or immediate euthanasia.
- All unexpected adverse events are reported immediately to the University Veterinarian, Chief Investigator and Facility Manager and an Unexpected Adverse Event Report completed and submitted to the Animal Ethics Officer.

**University of South Australia*- CLINICAL RECORD SHEET***

***Study Name: Development of kinase inhibitors in combination with other anti-cancer agents***

| cHIEF INVESTIGATOR: |  | | WORK PHONE: | |  | AFTER HOURS PHONE: |  |
| --- | --- | --- | --- | --- | --- | --- | --- |
| rESEARCHER: |  | | WORK PHONE: | |  | AFTER HOURS PHONE: |  |
| ETHICS NO: |  | | CAGE NO/ANIMAL ID: | |  | UNIVERSITY VETERINARIAN NAME/CONTACT |  |
| COMMENCEMENT  WEIGHT (g) |  | | 90% OF COMMENCEMENT WEIGHT: | |  | 85% OF COMMENCEMENT WEIGHT: |  |
| TREATMENT COMMENCEMENT DATE | | 6/3/2026 | | KINASE INHIBITOR/ DOSE/ROUTE | |  | |

|  |  | **1** | **2** | **3** | **4** | **5** | **6** | **7** |  |  |  |  |  |  |  |
| --- | --- | --- | --- | --- | --- | --- | --- | --- | --- | --- | --- | --- | --- | --- | --- |
| **DATE** | **TIME** | **Movement** | **Hunching** | **ruffled coat** | **Abnormal breathing** | **Skin tenting/ sunken eyes** | **Diarrhoea – bloody/profuse** | **Seizure/ erratic movement** | **TOTAL SCORE** | **Ulceration of tumour** | **BCS***  **X = Less than 2/5** | **Tumour volume** | **Weight (g)** | **COMMENTS**  (Record if Uni Vet or senior CAF technician were consulted) | **SIGN** |
|  |  |  |  |  |  |  |  |  |  |  |  |  |  |  |  |
|  |  |  |  |  |  |  |  |  |  |  |  |  |  |  |  |
|  |  |  |  |  |  |  |  |  |  |  |  |  |  |  |  |
|  |  |  |  |  |  |  |  |  |  |  |  |  |  |  |  |
|  |  |  |  |  |  |  |  |  |  |  |  |  |  |  |  |
|  |  |  |  |  |  |  |  |  |  |  |  |  |  |  |  |
|  |  |  |  |  |  |  |  |  |  |  |  |  |  |  |  |
|  |  |  |  |  |  |  |  |  |  |  |  |  |  |  |  |

See Coversheet for detailed descriptions of clinical criteria and actions and monitoring/weighing frequencies

**Actions for scores:**

**1** – Monitor daily; **cumulative 2** –Give soaked food and monitor twice daily, **2 for any one criterion** – Consult Uni Vet, follow advice; **3** – Consult Uni Vets, monitor 3 times daily; **4 or over** – Immediate HK.

**Ulceration of tumours:** Any sign of ulceration score – **1**; Consult Uni Vets and follow advice

**Tumour Volume**: ≥ 1500mm3- Consult Uni Vets and follow advice

**Weight loss:** 10% or greater -check sipper tube, give soaked food, consult senior technician, weighing daily and monitor twice daily; if persists for 48 hrs – consult Uni Vet or HK

**Weight loss:** 15% or greater–HK.

**Prolonged whole-body seizure/continuous erratic movement:** –HK **Any neurological signs:** – Consult Uni Vet

**ABSOLUTE HUMANE ENDPOINTS: laboured breathing; BCS < 2/5; a total score of 4; tumour volume = 2000mm^3^.**

**IF IN DOUBT OR CONCERNS CONTACT UNI VET**

*Refer to the ‘Extra CRS Information’ sheet for a description of body condition scoring.

**EXTRA CRS INFORMATION**

# **Supplementary Tables**

# **Table S1. List of primary antibodies used for western blot analysis.**

| **Antibody** | **Clone** | **Catalogue #** | **Host** | **Dilution** | **Molecular weight (KDa)** | **Supplier** |
| --- | --- | --- | --- | --- | --- | --- |
| **Topoisomerase II** | D10G9 | 12286 | Rabbit | 1:1000 | 190 | Cell Signaling Technology |
| **FOXM1** | D12D5 | 5436 | Rabbit | 1:1000 | 110 | Cell Signaling Technology |
| **Cyclin A2** | BF683 | 4656 | Mouse | 1:2000 | 55 | Cell Signaling Technology |
| **Cyclin B1** | - | 4138 | Rabbit | 1:1000 | 55 | Cell Signaling Technology |
| **Cyclin E2** | - | 4132 | Rabbit | 1:1000 | 48 | Cell Signaling Technology |
| **CDK2** | 78B2 | 2546 | Rabbit | 1:1000 | 33 | Cell Signaling Technology |
| **TK1** | - | 8960 | Rabbit | 1:1000 | 26 | Cell Signaling Technology |
| **pRB (S870)** | - | 9307 | Rabbit | 1:1000 | 110 | Cell Signaling Technology |
| **pRB (S807/811)** | - | 9308 | Rabbit | 1:1000 | 100 | Cell Signaling Technology |
| **AR** | ER179 | ab108341 | Rabbit | 1:2000 | 110 | Abcam |
| **PSA** | - | 10679-1-AP | Rabbit | 1:2000 | 29 | ProteinTech Group |
| **β-Tubulin** | - | 2146 | Rabbit | 1:1000 | 55 | Cell signalling Technology |
| **α-Tubulin** | - | T6074 | Mouse | 1:5000 | 55 | Merck / Sigma-Aldrich |

**Supplementary Table S2: Human cDNA primers used for qRT-PCR in this study. All primers were sourced from Merck.**

| **Name** | **Sequence (5’ -3’)** |
| --- | --- |
| *ACTB* Forward | GGCCAACCGCGAGAAGAT |
| *ACTB* Reverse | ATCACGATGCCAGTGGTACG |
| *AR* Forward | CCTGGCTTCCGCAACTTACAC |
| *AR* Reverse | GGACTTGTGCATGCGGTACTCA |
| *FOXM1* Forward | TCTGCCAATGGCAAGGTCTCCT |
| *FOXM1* Reverse | CTGGATTCGGTCGTTTCTGCTG |
| *GAPDH* Forward | TGCACCACCAACTGCTTAGC |
| *GAPDH* Reverse | GGCATGGACTGTGGTCATGAG |
| *KLK3* Forward | ACCAGAGGAGTTCTTGACCCCAAA |
| *KLK3* Reverse | CCCCAGAATCACCCGAGCAG |

# **Table S3: Statistical analysis of tumour volume ratio among treatment groups in LNCaP and PC3 xenograft models.**

| **Model** | **Comparison groups** | **Measurement days^#^** | | | | | | | | | | |
| --- | --- | --- | --- | --- | --- | --- | --- | --- | --- | --- | --- | --- |
|  |  | **1** | **3** | **5** | **7** | **9** | **11** | **13** | **15** | **17** | **19** | **21** |
| **LNCaP** | Vehicle *vs* AU2-94 75 mg/kg PO BID | >0.9999 | 0.9449 | 0.0509 | 0.0038 | 0.0002 | <0.0001 | <0.0001 | <0.0001 | <0.0001 | <0.0001 | <0.0001 |
| **PC3 (Experiment #1)** | Vehicle *vs* AU2-94 25 mg/kg PO BID | >0.9999 | 0.9642 | 0.3005 | 0.1863 | 0.0163 | 0.0031 | 0.0003 | <0.0001 | <0.0001 | <0.0001 | <0.0001 |
|  | Vehicle *vs* Atirmociclib 23 mg/kg PO BID | >0.9999 | 0.9636 | 0.5943 | 0.6015 | 0.3163 | 0.1152 | 0.0204 | 0.0015 | 0.0001 | <0.0001 | <0.0001 |
|  | AU2-94 25 mg/kg PO BID *vs* Atirmociclib 23 mg/kg PO BID | 0.9999 | >0.9999 | 0.8667 | 0.7068 | 0.3851 | 0.3941 | 0.4268 | 0.1632 | 0.3027 | 0.2309 | 0.2309 |
| **PC3 (Experiment #2)** | Vehicle vs. AU2-94 15 mg/kg, PO BID | >0.9999 | 0.7256 | 0.2185 | 0.0436 | 0.0012 | <0.0001 | <0.0001 | <0.0001 | <0.0001 | <0.0001 | <0.0001 |
|  | Vehicle vs. AU2-94 37.5 mg/kg, PO BID | >0.9999 | 0.5012 | 0.0436 | 0.0048 | <0.0001 | <0.0001 | <0.0001 | <0.0001 | <0.0001 | <0.0001 | <0.0001 |
|  | Vehicle vs. AU2-94 50 mg/kg, PO BID | >0.9999 | 0.14 | 0.0013 | <0.0001 | <0.0001 | <0.0001 | <0.0001 | <0.0001 | <0.0001 | <0.0001 | <0.0001 |
|  | Vehicle vs. Docetaxel 10 mg/kg, IP QW | >0.9999 | 0.6028 | 0.2873 | 0.2847 | 0.0258 | <0.0001 | <0.0001 | <0.0001 | <0.0001 | <0.0001 | <0.0001 |
|  | Vehicle vs. AU2-94 37.5 mg/kg + Docetaxel 10 mg/kg | >0.9999 | 0.1497 | 0.0105 | 0.0007 | <0.0001 | <0.0001 | <0.0001 | <0.0001 | <0.0001 | <0.0001 | <0.0001 |
|  | Vehicle vs. Ribociclib 37.5 mg/kg, PO BID | >0.9999 | 0.7249 | 0.6264 | 0.4999 | 0.2175 | <0.0001 | <0.0001 | <0.0001 | <0.0001 | <0.0001 | <0.0001 |
|  | AU2-94 15 mg/kg, PO BID vs. AU2-94 37.5 mg/kg, PO BID | >0.9999 | 0.9999 | 0.9951 | 0.9941 | 0.9933 | 0.8383 | 0.1416 | 0.0566 | 0.0001 | <0.0001 | <0.0001 |
|  | AU2-94 15 mg/kg, PO BID vs. AU2-94 50 mg/kg, PO BID | >0.9999 | 0.9472 | 0.6481 | 0.6703 | 0.3137 | 0.0015 | <0.0001 | <0.0001 | <0.0001 | <0.0001 | <0.0001 |
|  | AU2-94 15 mg/kg, PO BID vs. Docetaxel 10 mg/kg, IP QW | >0.9999 | >0.9999 | >0.9999 | 0.9866 | 0.9794 | 0.9822 | 0.2696 | 0.017 | <0.0001 | <0.0001 | <0.0001 |
|  | AU2-94 15 mg/kg, PO BID vs. Ribociclib 37.5 mg/kg, PO BID | >0.9999 | >0.9999 | 0.9998 | 0.9907 | 0.9188 | >0.9999 | 0.9866 | 0.7762 | 0.0943 | 0.0061 | <0.0001 |
|  | AU2-94 37.5 mg/kg, PO BID vs. AU2-94 50 mg/kg, PO BID | >0.9999 | 0.9931 | 0.9528 | 0.9646 | 0.7548 | 0.1137 | 0.0691 | 0.0168 | 0.0013 | 0.0002 | <0.0001 |
|  | AU2-94 37.5 mg/kg, PO BID vs. Docetaxel 10 mg/kg, IP QW | >0.9999 | >0.9999 | 0.9861 | 0.7717 | 0.7226 | 0.9988 | >0.9999 | 0.9997 | 0.9997 | 0.0926 | 0.1234 |
|  | AU2-94 37.5 mg/kg, PO BID vs. AU2-94 37.5 mg/kg + DTX 10 mg/kg | >0.9999 | 0.9945 | 0.9994 | 0.9988 | 0.9861 | 0.4785 | 0.1323 | 0.0049 | <0.0001 | <0.0001 | <0.0001 |
|  | AU2-94 37.5 mg/kg, PO BID vs. Ribociclib 37.5 mg/kg, PO BID | >0.9999 | >0.9999 | 0.9697 | 0.8461 | 0.6001 | 0.8754 | 0.7849 | 0.9348 | 0.8851 | 0.9977 | >0.9999 |
|  | AU2-94 50 mg/kg, PO BID vs. Docetaxel 10 mg/kg, IP QW | >0.9999 | 0.9806 | 0.5536 | 0.1996 | 0.0418 | 0.0282 | 0.0287 | 0.0559 | 0.006 | 0.5864 | 0.3702 |
|  | AU2-94 50 mg/kg, PO BID vs. Ribociclib 37.5 mg/kg, PO BID | >0.9999 | 0.9934 | 0.5566 | 0.3347 | 0.0432 | 0.0078 | 0.0021 | 0.0017 | <0.0001 | 0.0002 | 0.0007 |
|  | Docetaxel 10 mg/kg, IP QW vs. AU2-94 37.5 mg/kg + DTX 10 mg/kg | >0.9999 | 0.9838 | 0.8795 | 0.4435 | 0.234 | 0.1975 | 0.0606 | 0.0193 | 0.0002 | 0.0015 | <0.0001 |
|  | Docetaxel 10 mg/kg, IP QW vs. Ribociclib 37.5 mg/kg, PO BID | >0.9999 | >0.9999 | >0.9999 | >0.9999 | 0.9997 | 0.9838 | 0.9061 | 0.791 | 0.7039 | 0.0542 | 0.2182 |

^#^Statistical analysis using two-way ANOVA method.

# **Table S4: Statistical analysis of body weight changes among treatment groups in LNCaP and PC3 xenograft models.**

| **Model** | **Comparison groups** | **Measurement days^#^** | | | | | | | | | | | | | | | | | | | |  |
| --- | --- | --- | --- | --- | --- | --- | --- | --- | --- | --- | --- | --- | --- | --- | --- | --- | --- | --- | --- | --- | --- | --- |
|  |  | **1** | **2** | **3** | **4** | **5** | **6** | **7** | **8** | **9** | **10** | **11** | **12** | **13** | **14** | **15** | **16** | **17** | **18** | **19** | **20** | **21** |
| **LNCaP** | Vehicle *vs* AU2-94 75 mg/kg, PO BID | 0.994 | 0.996 | 0.874 | 0.674 | 0.855 | 0.813 | 0.979 | 0.921 | 0.802 | 0.835 | 0.802 | 0.802 | 0.778 | 0.751 | 0.855 | 0.548 | 0.674 | 0.715 | 0.728 | 0.845 | 0.728 |
| **PC3 (Experiment #1)** | Vehicle *vs* AU2-94 25 mg/kg, PO BID | >0.999 | 0.961 | 0.725 | 0.630 | 0.271 | 0.079 | 0.169 | 0.187 | 0.011 | 0.009 | 0.001 | <0.0001 | <0.0001 | <0.0001 | <0.0001 | 0.0001 | <0.0001 | 0.0002 | 0.0001 | 0.0001 | 0.001 |
|  | Vehicle *vs* Atirmociclib 23 mg/kg, PO BID | >0.999 | 0.946 | 0.941 | 0.997 | 0.832 | 0.648 | 0.716 | 0.988 | 0.999 | 0.876 | 0.349 | 0.644 | 0.961 | 0.361 | 0.144 | 0.828 | 0.179 | 0.295 | 0.468 | 0.780 | 0.832 |
|  | AU2-94 25 mg/kg, PO BID *vs* Atirmociclib 23 mg/kg, PO BID | >0.999 | 0.998 | 0.902 | 0.676 | 0.598 | 0.413 | 0.562 | 0.243 | 0.010 | 0.037 | 0.067 | 0.0006 | 0.0003 | <0.0001 | 0.001 | 0.001 | 0.0002 | 0.032 | 0.009 | 0.002 | 0.004 |
| **PC3 (Experiment #2)** | Vehicle vs. AU2-94 15 mg/kg, PO BID | >0.999 | 0.8997 | 0.5711 | 0.6609 | 0.6609 | 0.7197 | 0.194 | 0.5464 | 0.1784 | 0.2469 | 0.9604 | 0.9586 | 0.979 | 0.9945 | 0.798 | 0.4901 | 0.1791 | 0.9718 | 0.7499 | 0.8038 | 0.5829 |
|  | Vehicle vs. AU2-94 37.5 mg/kg, PO BID | >0.999 | 0.9883 | 0.1435 | 0.2533 | 0.2533 | 0.0226 | 0.4836 | 0.372 | 0.4061 | 0.0304 | 0.0057 | 0.0043 | 0.0084 | 0.0057 | 0.0043 | 0.0047 | 0.0013 | <0.0001 | 0.003 | 0.0011 | <0.0001 |
|  | Vehicle vs. AU2-94 50 mg/kg, PO BID | >0.999 | 0.8355 | 0.016 | 0.0027 | 0.0027 | <0.0001 | 0.0001 | 0.0001 | 0.0007 | 0.0001 | <0.0001 | <0.0001 | <0.0001 | <0.0001 | <0.0001 | 0.0003 | <0.0001 | <0.0001 | <0.0001 | 0.0004 | <0.0001 |
|  | Vehicle vs. Docetaxel 10 mg/kg, IP QW | >0.999 | 0.8234 | 0.6906 | 0.9714 | 0.9714 | 0.2313 | 0.6857 | >0.9999 | 0.4283 | 0.989 | 0.372 | 0.045 | 0.1107 | 0.1967 | 0.4836 | 0.4948 | 0.6079 | 0.9183 | 0.3611 | 0.6678 | 0.8487 |
|  | Vehicle vs. AU2-94 37.5 mg/kg + Docetaxel 10 mg/kg | >0.999 | 0.8767 | 0.3128 | 0.1007 | 0.1007 | 0.024 | 0.2533 | 0.5415 | 0.0102 | 0.0008 | <0.0001 | <0.0001 | <0.0001 | <0.0001 | <0.0001 | <0.0001 | <0.0001 | <0.0001 | <0.0001 | <0.0001 | <0.0001 |
|  | Vehicle vs. Ribociclib 37.5 mg/kg, PO BID | >0.999 | 0.9993 | >0.9999 | 0.7543 | 0.7543 | 0.9745 | 0.9605 | 0.6521 | 0.9956 | 0.9672 | 0.9973 | 0.9995 | >0.9999 | >0.9999 | 0.8923 | 0.9749 | 0.9986 | >0.9999 | >0.9999 | >0.9999 | >0.9999 |

^#^Statistical analysis using two-way ANOVA method.

**Supplementary Table S5. Number of genes differentially expressed (adjusted p ≤ 0.05, log2 fold-change ≥ 1) with indicated treatments.**

| **24 h treatment** | **Treatment group** | | | | |
| --- | --- | --- | --- | --- | --- |
|  | **AU2-94 1μM** | **AU2-94 2μM** | **Atirmociclib 1μM** | **Atirmociclib 1μM** | **Ribociclib 2μM** |
| **TOTAL** | 411 | 556 | 676 | 694 | 593 |
| **UPREGULATED** | 123 | 180 | 143 | 103 | 126 |
| **DOWNREGULATED** | 288 | 376 | 533 | 591 | 467 |
| **72 h treatment** | **Treatment group** | | | | |
|  | **AU2-94 1μM** | **AU2-94 2μM** | **Atirmociclib 1μM** | **Atirmociclib 1μM** | **Ribociclib 2μM** |
| **TOTAL** | 300 | 275 | 645 | 789 | 364 |
| **UPREGULATED** | 235 | 215 | 349 | 387 | 230 |
| **DOWNREGULATED** | 65 | 60 | 296 | 402 | 134 |

# **Supplementary Figures**

**Supplementary Figure S1. The effect of CDK4 inhibition on** clonogenic growth of prostate cancer cells. Prostate cancer cell lines (C4-2B, 22Rv1, PC3, LNCaP, DU145) were treated with increasing concentrations of AU2-94 and atirmociclib for 10 days. Media was refreshed every 72 hours to maintain drug exposure according to the treatment schedule. After 10 days, media were removed and colonies were fixed and stained with crystal violet solution. Colony images were acquired, and the number of colonies was quantified using ImageJ software. Representative images from three independent experiments are shown.

**Supplementary Figure S2. Quantification of western blot analysis from tumour samples derived from LNCaP (A) and PC3 (B) xenograft models.** In the LNCaP model, mice were treated with AU2-94 (75 mg/kg, BID PO), while in the PC3 model mice received AU2-94 (25 mg/kg, BID PO) or atirmociclib (23 mg/kg, BID PO) for 21 days. Tumours were harvested 12 h after the final dose on day 21, lysed, and total protein extracted. Band intensities were quantified using ImageJ software and normalized to β-tubulin. Data are presented as mean ± SD from three mice per treatment group.

**Supplementary Figure S3. CDK4 inhibition stimulates AR activity in prostate cancer. (A)** Commonly used AR activity gene sets were evaluated by gene set enrichment analysis. Gene sets significantly altered (q < 0.1) in at least one treatment are shown. NES, normalised enrichment score. **(B)** AU2-94-stimulated induction of *KLK3* is reversed by enzalutamide co-treatment, as evaluated using qRT-PCR. *GAPDH* and *ACTB* were used for normalization. **(C)** As for B, except PSA (the protein product of *KLK3*) was measured using Western blotting. Western blots on left were generated using a pool of 3 biological replicates for each treatment group. The graph on right shows relative levels of PSA normalised to total protein, determined from a separate Western blot in which all 3 replicates were measured individually (not shown). For B-C, error bars are ± SEM and statistical differences between groups were assessed using ordinary one-way ANOVA and Dunnett’s multiple comparisons testing (b, p ≤ 0.01; d, p ≤ 0.0001).
